# ﻿Review of the genus *Salicarus* (Hemiptera, Heteroptera, Miridae)

**DOI:** 10.3897/zookeys.1211.129660

**Published:** 2024-09-02

**Authors:** Fedor V. Konstantinov, Reza Hosseini

**Affiliations:** 1 Zoological Institute, Russian Academy of Sciences, Universitetskaya nab. 1, St. Petersburg 199034, Russia Zoological Institute, Russian Academy of Sciences St. Petersburg Russia; 2 National Museum of Natural History, Bulgarian Academy of Sciences, 1 Tsar Osvoboditel Blvd, 1000 Sofia, Bulgaria National Museum of Natural History, Bulgarian Academy of Sciences Sofia Bulgaria; 3 Department of Plant Protection, Faculty of Agricultural Sciences, University of Guilan, Rasht, 41635–1314, Iran University of Guilan Rasht Iran

**Keywords:** Distribution, hosts, new synonymy, Palearctic, phylogeny, taxonomy

## Abstract

The genus *Salicarus* Kerzhner, 1962 is revised, with differential diagnoses and redescriptions provided for nine species. Three distinct species groups were recognized within the genus: *S.nitidus* group (*S.cavinotum*, *S.genistae*, *S.nitidus*, *S.perpusillus*), *S.roseri* group (*S.concinnus*, *S.roseri*, *S.urnammu*), and *S.fulvicornis* group (*S.halimodendri*, *S.fulvicornis*). A key to species, illustrations of dorsal habitus, male and female genitalia, and selected diagnostic structures are included. Additionally, available host information and distributional records are summarized. *Phoenicocorisqiliananus* Zheng, 1996 is synonymized with *Salicarushalimodendri* V. G. Putshkov, 1977.

## ﻿Introduction

*Salicarus* Kerzhner, 1962 belongs to the subfamily Phylinae of the hyperdiverse family Miridae, or plant bugs, the second largest family of insects with incomplete metamorphosis (Cassis et al. 2007). Phylines are, in turn, the second largest subfamily of plant bugs, predominantly host specific, with many taxa still lacking adequate diagnoses. The genus has a convoluted taxonomic history, with most species now treated within *Salicarus* being previously placed in several different genera. Kerzhner (1962) erected the genus *Salicarus* for the single species, *S.roseri* (Herrich-Schaeffer, 1839), in an effort to create monophyletic groupings within the wide array of species previously assigned to *Sthenarus* Fieber, 1858. Wagner (1975a) considered *Salicarus* (incorrectly spelt *Salicarius*) as a subgenus within *Sthenarus* but included a wide assemblage of species, all of which except the type species were subsequently transferred to *Campylomma* Reuter, 1878 (Kerzhner and Matocq 1997; Konstantinov 2023) or *Psallus* Fieber, 1858 (Wagner 1975b). Putshkov (1977) described two new species of *Salicarus* and updated the concept of the genus without commenting on the Mediterranean species. Stonedahl (1990) and Schwartz and Stonedahl (2004) published revisions of the genera *Atractotomus* Fieber, 1858 and *Phoenicocoris* Reuter, 1875, respectively, providing important insights into the taxonomy of these and related genera, including *Salicarus* and *Heterocapillus* Wagner, 1960. The latter genus was considered non-monophyletic and consisting of several unrelated groups of species (Stonedahl 1990; Konstantinov 2008). Konstantinov (2023) performed a morphology-based phylogenetic analysis of the group in question, showing the low taxonomic utility of many characters of external morphology traditionally used in the generic taxonomy of these genera. He updated the diagnosis and species composition of *Salicarus*, particularly by transferring to it four species previously assigned to *Heterocapillus*.

The present paper serves as a supplement to the mentioned revision by Konstantinov (2023) and aims to summarize current knowledge about *Salicarus*, including species delimitation, distributional ranges, and host plant associations. Consequently, a key to all nine species and standardized species redescriptions with detailed illustrations are provided.

## ﻿Materials and methods

### ﻿Microscopy, illustrations, and terminology

Observations, measurements, and habitus images were made using a Zeiss Stemi 508 stereomicroscope equipped with a Canon EOS 2000D digital SLR camera. Partially focused images of each specimen or structure were stacked using Helicon Focus software. Images of the selected structures, including male and female genitalia, were taken with a Keyence VHX–500F digital microscope at the University of Hamburg. Illustrated structures were macerated in potassium hydroxide, cleared in distilled water, and then transferred to glycerin jelly for proper orientation. Scanning electron micrographs of selected structures were taken using a Quanta 250 scanning electron microscope.

Unless otherwise stated, all measurements are in millimeters. Measurements shown in Table 1 include body length, clypeus to apex of cuneus length, width of head, interocular distance, length of antennal segments I and II, and pronotum length and width. The morphological terminology follows Schuh and Weirauch (2020), except for genitalia, which follows Konstantinov (2003, 2019) for males and Schwartz (2011) for females.

**Table 1.** Measurements (mm). Abbreviations. Cun–Clyp – distance between apex of clypeus and apex of corium in dorsal view, Head Length – distance between apex oSf clypeus and the highest point of vertex, AntSeg1, AntSeg2 – length of antennal segments I and II, InterOcDi – width of vertex between inner margins of eyes in dorsal view.

Species	Length						Width			
		Body	Cun–Clyp	Pronotum	Tibia3	AntSeg2	Head	InterOcDi		Pronotum
***Salicaruscavinotum***										
♂♂ (n = 5)	**Mean**	**2.16**	**1.86**	**0.37**	**0.98**	**0.49**	**0.65**		**0.36**	**0.83**
	SD	0.25	0.27	0.03	0.05	0.03	0.03		0.02	0.05
	Range	0.60	0.70	0.06	0.14	0.07	0.07		0.05	0.10
	Min	2.00	1.60	0.34	0.90	0.46	0.61		0.34	0.78
	Max	2.60	2.30	0.40	1.04	0.53	0.68		0.39	0.88
♀♀ (n = 5)	**Mean**	**2.15**	**1.91**	**0.39**	**1.02**	**0.49**	**0.68**		**0.40**	**0.89**
	SD	0.09	0.08	0.03	0.07	0.03	0.03		0.01	0.04
	Range	0.23	0.22	0.09	0.15	0.07	0.06		0.04	0.09
	Min	2.05	1.83	0.35	0.95	0.45	0.65		0.38	0.85
	Max	2.28	2.04	0.44	1.10	0.52	0.71		0.41	0.94
***Salicarusconcinnus***										
♂♂ (n = 5)	**Mean**	**3.21**	**2.78**	**0.62**	**1.51**	**0.71**	**0.80**		**0.42**	**1.25**
	SD	0.33	0.21	0.03	0.04	0.04	0.02		0.01	0.07
	Range	0.75	0.50	0.08	0.13	0.10	0.05		0.01	0.18
	Min	2.95	2.63	0.58	1.45	0.65	0.78		0.41	1.18
	Max	3.70	3.13	0.65	1.58	0.75	0.83		0.43	1.35
♀♀ (n = 5)	**Mean**	**3.24**	**2.79**	**0.59**	**1.56**	**0.71**	**0.81**		**0.44**	**1.25**
	SD	0.18	0.11	0.06	0.11	0.09	0.03		0.01	0.09
	Range	0.45	0.25	0.15	0.20	0.20	0.06		0.03	0.23
	Min	3.00	2.70	0.53	1.45	0.65	0.79		0.43	1.15
	Max	3.45	2.95	0.68	1.65	0.85	0.85		0.45	1.38
***Salicarusfulvicornis***										
♂♂ (n = 5)	**Mean**	**3.82**	**3.08**	**0.57**	**1.54**	**0.85**	**0.80**		**0.40**	**1.20**
	SD	0.10	0.07	0.05	0.06	0.05	0.02		0.00	0.12
	Range	0.25	0.15	0.10	0.18	0.10	0.05		0.00	0.30
	Min	3.70	3.00	0.53	1.45	0.80	0.78		0.40	1.00
	Max	3.95	3.15	0.63	1.63	0.90	0.83		0.40	1.30
♀♀ (n = 5)	**Mean**	**3.29**	**2.82**	**0.53**	**1.41**	**0.73**	**0.84**		**0.45**	**1.18**
	SD	0.14	0.10	0.02	0.01	0.05	0.02		0.02	0.05
	Range	0.35	0.25	0.05	0.03	0.10	0.04		0.05	0.13
	Min	3.15	2.70	0.50	1.40	0.68	0.83		0.43	1.13
	Max	3.50	2.95	0.55	1.43	0.78	0.86		0.48	1.25
***Salicarusgenistae***										
♂♂ (n = 5)	Mean	**2.36**	**2.10**	**0.46**	**1.18**	**0.58**	**0.75**		**0.39**	**1.04**
	SD	0.11	0.08	0.02	0.03	0.02	0.02		0.01	0.01
	Range	0.28	0.22	0.04	0.06	0.04	0.05		0.02	0.04
	Min	2.20	2.00	0.44	1.14	0.56	0.72		0.38	1.02
	Max	2.48	2.22	0.48	1.20	0.60	0.77		0.40	1.06
♀♀ (n = 5)	Mean	**2.72**	**2.43**	**0.49**	**1.27**	**0.59**	**0.79**		**0.43**	**1.06**
	SD	0.09	0.10	0.01	0.03	0.03	0.02		0.01	0.03
	Range	0.20	0.24	0.03	0.08	0.08	0.05		0.02	0.06
	Min	2.60	2.30	0.47	1.22	0.54	0.76		0.42	1.04
	Max	2.80	2.54	0.50	1.30	0.62	0.81		0.44	1.10
***Salicarushalimodendri***										
♂♂ (n = 5)	**Mean**	**3.71**	**3.11**	**0.57**	**1.42**	**0.78**	**0.89**		**0.45**	**1.32**
	SD	0.17	0.11	0.05	0.02	0.02	0.03		0.02	0.05
	Range	0.33	0.25	0.10	0.05	0.05	0.05		0.05	0.13
	Min	3.55	2.95	0.50	1.40	0.75	0.85		0.43	1.25
	Max	3.88	3.20	0.60	1.45	0.80	0.90		0.48	1.38
♀♀ (n = 5)	**Mean**	**3.34**	**2.96**	**0.58**	**1.41**	**0.69**	**0.92**		**0.49**	**1.36**
	SD	0.15	0.12	0.02	0.03	0.03	0.03		0.01	0.03
	Range	0.35	0.33	0.05	0.08	0.08	0.08		0.03	0.08
	Min	3.13	2.83	0.55	1.38	0.65	0.88		0.48	1.33
	Max	3.48	3.15	0.60	1.45	0.73	0.95		0.50	1.40
***Salicarusnitidus***										
♂♂ (n = 2)	Min	2.30	2.00	0.40	1.06	0.52	0.74		0.40	0.94
	Max	2.60	2.28	0.46	1.22	0.53	0.75		0.42	0.96
♀♀ (n = 2)	Min	2.18	1.98	0.42	1.07	0.56	0.70		0.40	0.96
	Max	2.40	2.16	0.44	1.14	0.57	0.74		0.40	0.98
***Salicarusperpusillus***										
♂♂ (n = 3)	**Mean**	**2.24**	**1.88**	**0.40**	**1.09**	**0.50**	**0.69**		**0.38**	**0.87**
	Min	2.14	1.84	0.38	1.02	0.49	0.66		0.37	0.84
	Max	2.37	1.95	0.41	1.14	0.52	0.71		0.39	0.88
♀♀ (n = 5)	**Mean**	**2.27**	**1.97**	**0.42**	**1.04**	**0.53**	**0.70**		**0.39**	**0.90**
	SD	0.11	0.14	0.02	0.02	0.01	0.03		0.01	0.03
	Range	0.24	0.33	0.04	0.04	0.03	0.07		0.02	0.07
	Min	2.16	1.83	0.40	1.02	0.51	0.66		0.38	0.86
	Max	2.40	2.16	0.44	1.06	0.54	0.73		0.40	0.93
***Salicarusroseri***										
♂♂ (n = 5)	**Mean**	**3.80**	**3.31**	**0.68**	**1.65**	**0.74**	**0.86**		**0.41**	**1.40**
	SD	0.16	0.20	0.02	0.10	0.04	0.03		0.02	0.06
	Range	0.38	0.50	0.05	0.25	0.10	0.08		0.05	0.15
	Min	3.58	3.13	0.65	1.58	0.68	0.83		0.39	1.33
	Max	3.95	3.63	0.70	1.83	0.78	0.90		0.44	1.48
♀♀ (n = 5)	**Mean**	**3.76**	**3.25**	**0.67**	**1.61**	**0.71**	**0.89**		**0.41**	**1.42**
	SD	0.22	0.21	0.06	0.07	0.07	0.02		0.05	0.07
	Range	0.55	0.50	0.15	0.20	0.18	0.05		0.13	0.18
	Min	3.38	2.88	0.58	1.50	0.60	0.85		0.33	1.30
	Max	3.93	3.38	0.73	1.70	0.78	0.90		0.45	1.48
***Salicarusurnammu***										
♂♂ (n = 3)	**Mean**	**3.61**	**3.04**	**0.67**	**1.53**	**0.73**	**0.85**		**0.43**	**1.28**
	Min	3.45	2.88	0.63	1.50	0.70	0.83		0.43	1.25
	Max	3.75	3.20	0.73	1.55	0.75	0.89		0.43	1.35
♀♀ (n = 3)	**Mean**	**3.34**	**2.94**	**0.63**	**1.48**	**0.66**	**0.85**		**0.43**	**1.29**
	Min	3.20	2.83	0.60	1.45	0.60	0.83		0.40	1.25
	Max	3.45	3.08	0.65	1.53	0.70	0.88		0.45	1.38

### ﻿Specimens and collections

The material examined for this study is retained in the following collections:

**HNHM**Hungarian Natural History Museum, Budapest (András Orosz);

**NMWC**National Museum of Wales, Cardiff (Mike Wilson);

**SNSB**Zoologische Staatssammlung München (Tanja Kothe);

**UGNHM** Natural History Museum of the University of Guilan, Rasht;

**ZISP**Zoological Institute, Russian Academy of Sciences, St. Petersburg;

**ZMUH**Zoological Museum, University of Hamburg (Frank Wieland, Martin Husemann).

Bar code labels, uniquely identifying each specimen, were attached to all examined specimens listed in the “Material examined” sections. Further information, such as additional photographs of habitus and genitalia structures, georeferenced coordinates, specimens dissected, notes, and collecting methods, can be obtained from the Heteroptera Species Pages (http://research.amnh.org/pbi/heteropteraspeciespage/), which assembles available data from a specimen database (Konstantinov and Namyatova 2019). Refer to Suppl. material 1 for unique specimen identifiers of illustrated specimens. Original locality data are given in square brackets if it differs from currently existing toponyms.

## ﻿Taxonomic account

### ﻿ Salicarus

Taxon classificationAnimaliaHemipteraMiridae

Kerzhner, 1962

8B061A96-637F-58E8-B490-33BB54FE2DD2

Salicarus Kerzhner, 1962: 381. Type species by original designation: Capsusroseri Herrich-Schaeffer, 1838.
Salicarus : Putshkov (1977): 365 (revision); Kerzhner (1964): 996 (key, figures).
Salicarius [sic!]: Wagner (1975a): 99 (key, descriptions, figures).

#### Diagnosis.

**Figure 1.**
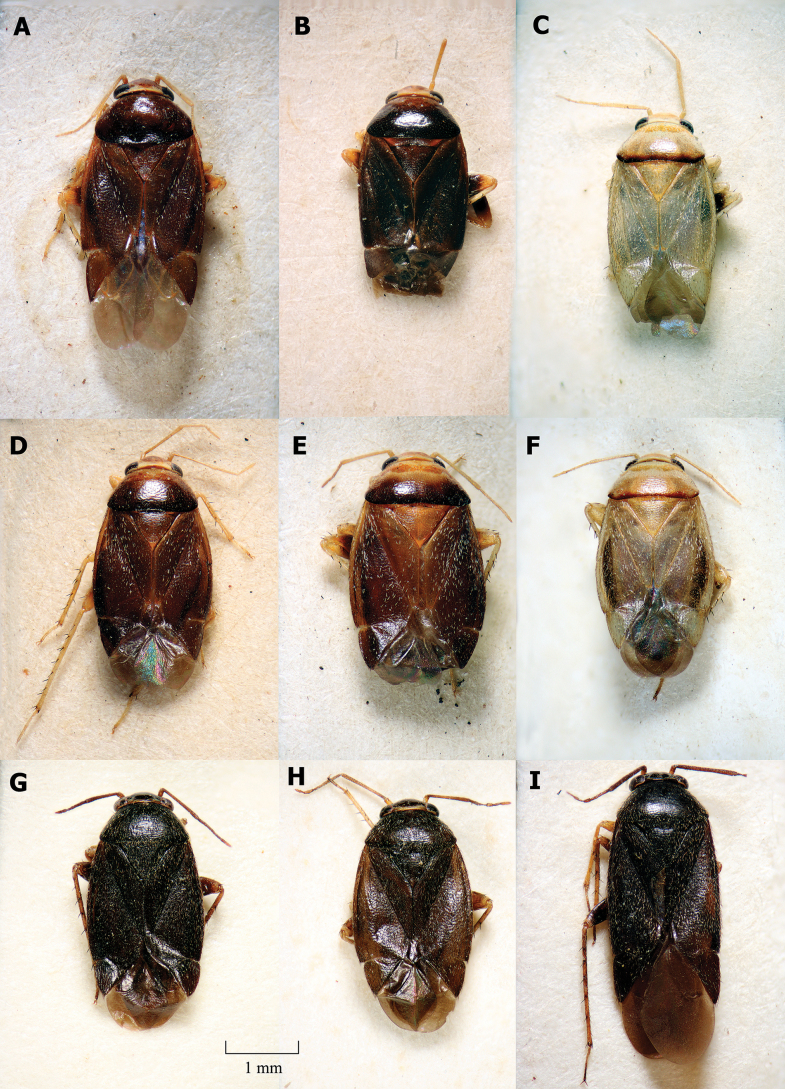
Dorsal habitus **A–C** ♂ paratype of *Salicarusconcinnus***D–F** ♀ paratype of *S.concinnus***G, H** ♀ *S.fulvicornis***I** ♂ *S.fulvicornis*.

**Figure 2.**
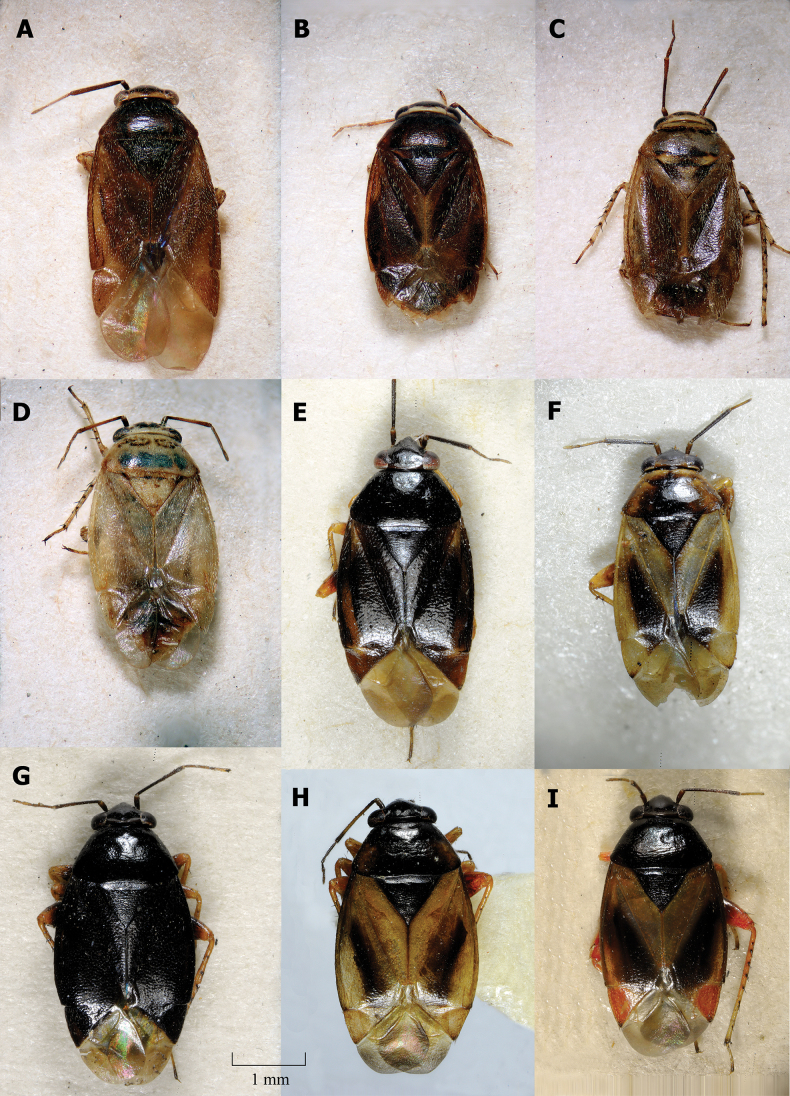
Dorsal habitus **A** ♂ *Salicarushalimodendri***B–D** ♀ *S.halimodendri***F, G** ♂ *S.roseri***H, I** ♀ *S.roseri*.

**Figure 3.**
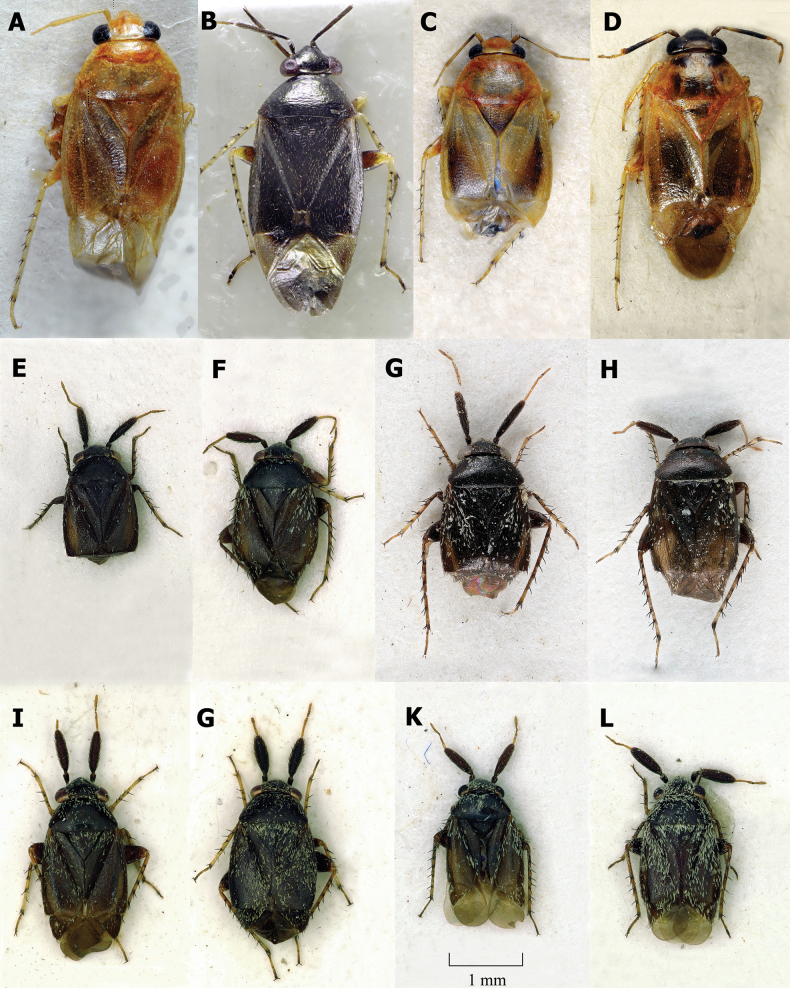
Dorsal habitus **A, B** ♂ *Salicarusurnammu***C, D** ♀ *S.urnammu***E** ♂ paratype of *S.cavinotum***F** ♀ paratype of *S.cavinotum***G** ♂ *S.genistae***H** ♀ *S.genistae***I** ♂ *S.nitidus***G** ♀ *S.nitidus***K** ♂ holotype of *S.perpusillus***L** ♀ *S.perpusillus*.

**Figure 4.**
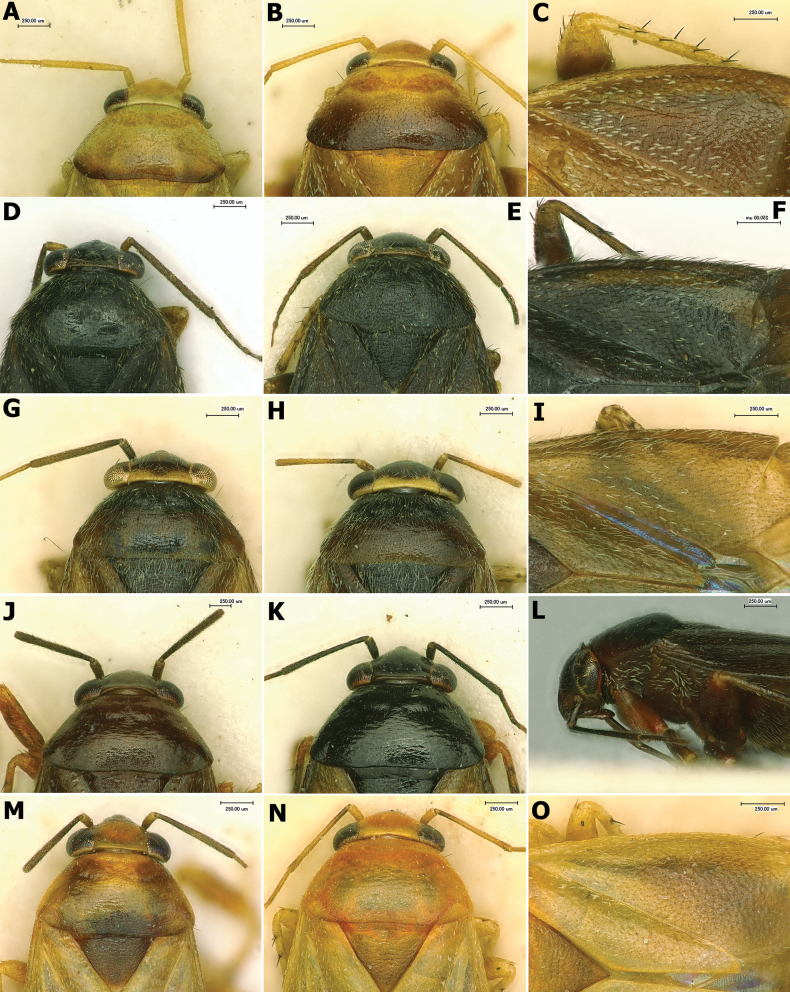
Head, pronotum, and vestiture **A–C***Salicarusconcinnus* in dorsal view: **A** ♂ paratype, head and pronotum **B** ♀ paratype, head and pronotum **C** ♀ paratype, vestiture on hemelytron **D–F***S.fulvicornis* in dorsal view: **D** ♂ head and pronotum **E** ♀ head and pronotum **F** ♂ vestiture on hemelytron **G–I***S.halimodendri* in dorsal view: **G** ♂ paratype **H** ♀ paratype **I** ♂ paratype, vestiture on hemelytron **J–L***S.roseri*: **J** ♂ head and pronotum **K** ♀ head and pronotum **L** head and thoracic pleura in lateral view **M–O***S.urnammu* in dorsal view: **M** ♂ head and pronotum **N** ♀ head and pronotum **O** ♀ vestiture on hemelytron.

**Figure 5.**
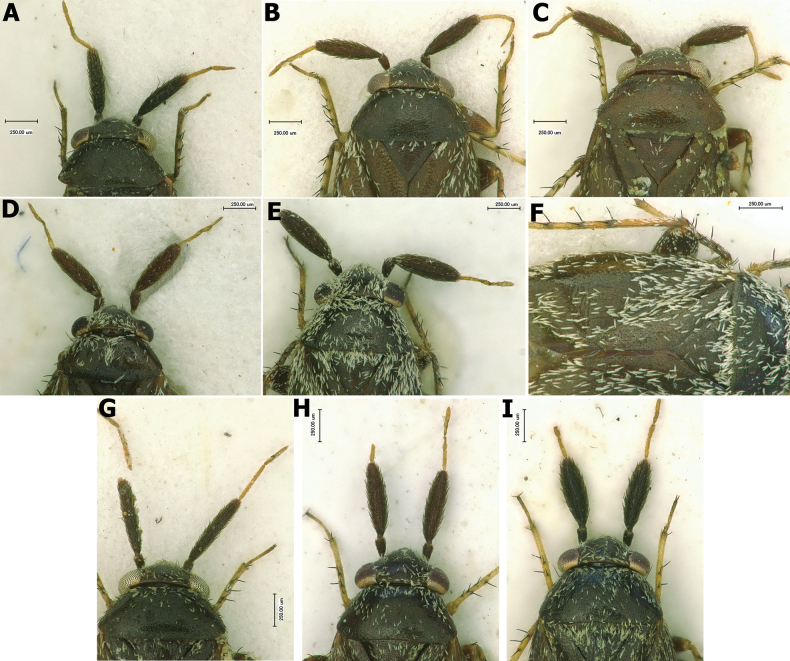
Head, pronotum, and vestiture **A, B** paratype of *Salicaruscavinotum*, head and pronotum in dorsal view: **A** ♂ **B** ♀ **C, G***S.genistae*, head and pronotum in dorsal view: **C** ♂ **G** ♀ **D–F** paratypes of *S.perpusillus*: **D** ♂ head and pronotum in dorsal view **E** ♀ head and pronotum in dorsal view **F** ♀ vestiture on dorsum and legs **H–I***S.nitidus*, head and pronotum in dorsal view: **H** ♂ **I** ♀.

**Figure 6.**
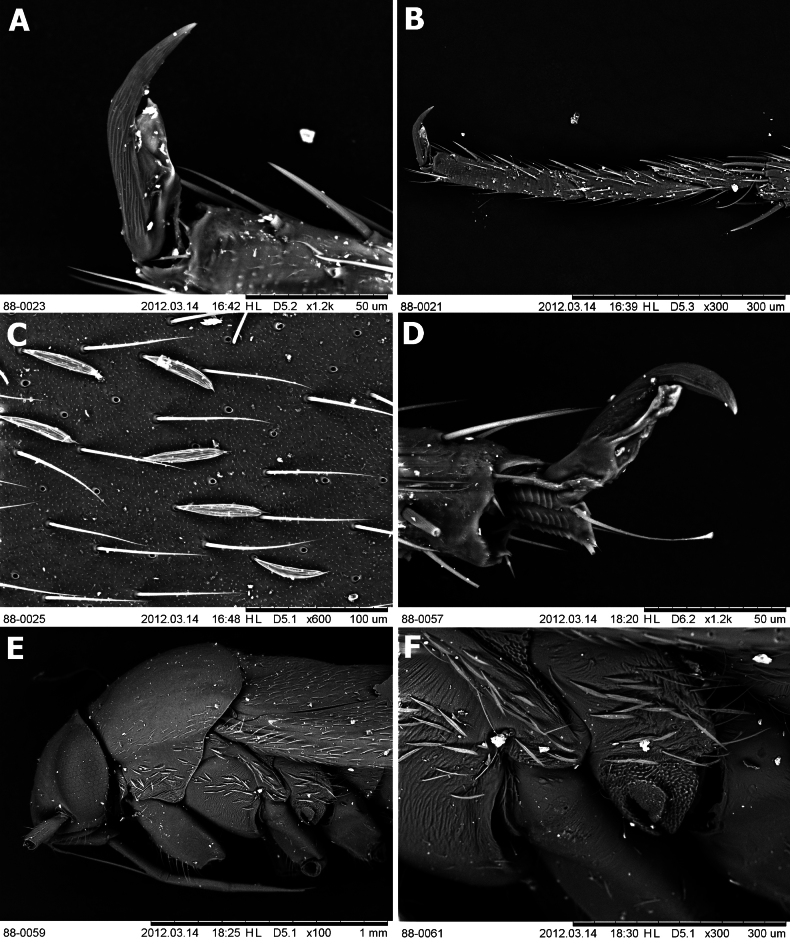
Scanning electron images of selected structures **A–C***Salicarusfulvicornis*: **A** pretarsus in lateral view **B** hind tarsus in lateral view **C** vestiture on hemelytron **D–F***S.roseri*: **D** pretarsus in lateral view **E** head and thoracic pleura in lateral view **F** metathoracic spiracle and scent gland evaporative area.

**Figure 7.**
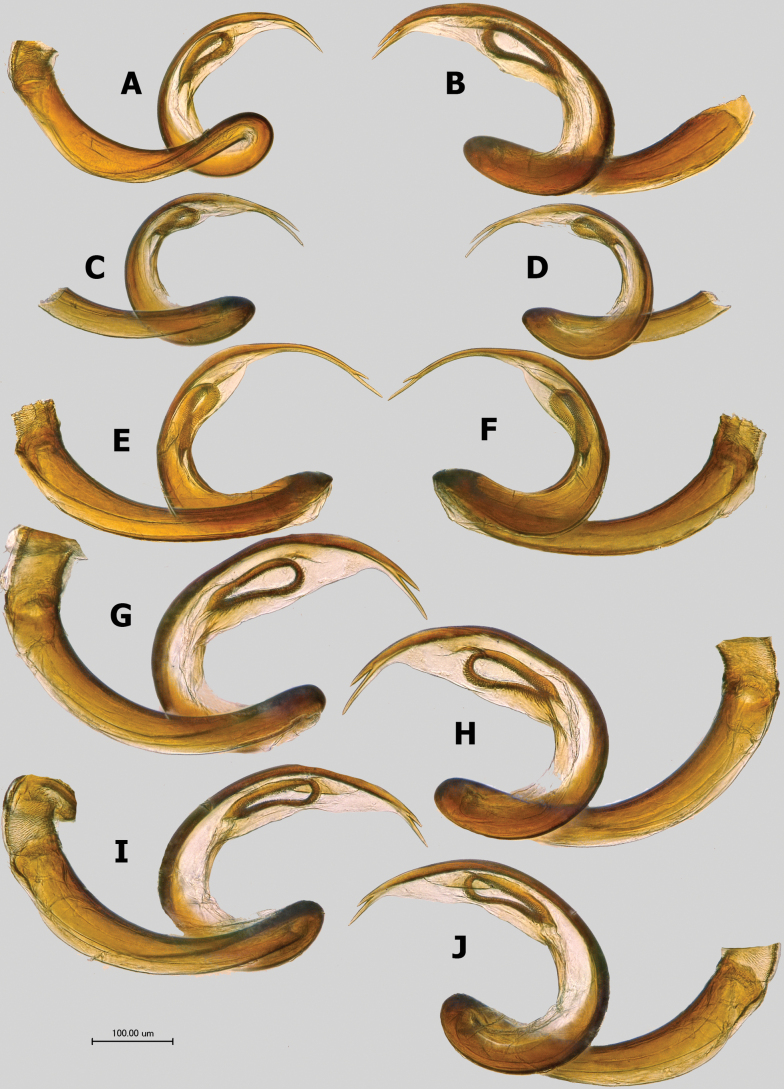
Vesica in left and right lateral views (left and right columns, respectively) **A, B** paratypes of *Salicarusconcinnus***C, D***S.fulvicornis***E, F** paratype of *S.halimodendri***G, H***S.roseri***I, J***S.urnammu*.

**Figure 8.**
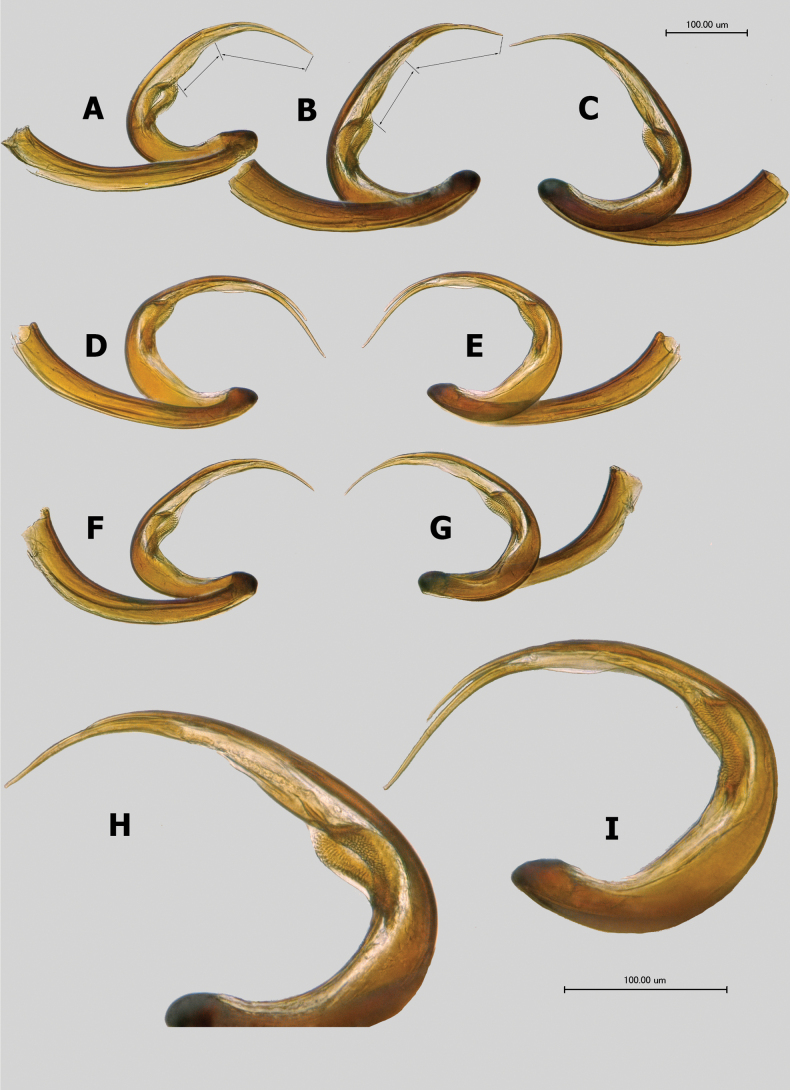
Vesica **A***Salicaruscavinotum*, paratype **B, C***S.genistae***D, E***S.nitidus***F, G***S.perpusillus*, paratype **H, I** apical part of vesica enlarged: **H***S.genistae***I***S.nitidus*.

Body broadly oval, with short appendages (Figs 1–3); head vertical, clypeus barely visible in dorsal view, posterior margin of vertex attenuate, covering anterior margin of pronotum (Figs 4, 5, 6E); dorsum and/or thoracic pleura clothed with scale-like setae and simple setae (Figs 4, 5, 6C); parempodium apically spatulate; pulvillum small, not reaching midpoint of claw (Fig. 6A, D); vesica large, strongly coiled at middle, apically with two long and thin, gradually tapering blades tightly fused along almost entire length (Figs 7A–D, 8), or slightly dilate apically (Fig. 7G–J); secondary gonopore large, located close to middle of vesica, equipped with gonopore sclerite; vestibulum of bursa copulatrix S-shaped, contrastingly long and thin (Fig. 10). Refer to Konstantinov (2023) for additional discussion.

#### Redescription.

**Male. *Coloration*.** Dorsum and venter uniformly chestnut to dark brown with whitish antennal segments III and IV in *S.nitidus* species group (Fig. 3E–L), in other species ranging from uniformly dark brown to pale yellow, often exhibiting significant polymorphism within a species (Figs 1, 2).

***Surface and vestiture*.** Dorsum shiny to moderately shiny, head and pronotum usually smooth, scutellum and hemelytron weakly rugose. Vestiture composed of dense simple setae intermixed with one of two types of scale-like setae: wide, apically serrate, densely covering dorsum, thoracic pleura, abdomen, and sometimes appendages (*S.nitidus* species group, Fig. 5) or narrow, apically acuminate, always located on thoracic pleura and usually also on dorsum or hemelytron only (other species, Figs 4, 6C). Thoracic pleurites devoid of simple setae and densely clothed exclusively with scale-like setae located above metathoracic scent gland evaporatory area, with no vestiture in ventral half (Fig. 6E). Simple setae usually adpressed, sometimes semierect, ranging from as long as to almost twice as long as scale-like setae; in addition, in *S.nitidus* species group, pronotum laterally and hemelytron proximally with robust, dark, contrastingly long, erect to semierect bristle-like simple setae. Appendages with simple, adpressed to semierect, usually pale setae; antennal segments I and II in *S.nitidus* species group with contrastingly dense, dark and robust simple setae (Fig. 5); head and fore coxa ventrally with contrastingly long simple silver setae; pronotum with a pair of black erect bristle-like setae at anterior corners; femora with several similar black setae dorso-apically; tibial spines dark brown to black.

***Structure*.** Body elongate-oval to oval, total length 2.0–4.0. ***Head***: Flattened and strongly sloping, barely protruding beyond anterior margin of eyes (Figs 4, 5, 6E); eyes occupying almost entire height of head in lateral view, posterolateral margins of eyes contiguous with anterolateral margins of pronotum; vertex flat, with attenuate posterior margin covering anterior margin of pronotum, frons vertical, clypeus not visible or barely visible in dorsal view; antenna inserted near ventral margin of eye; segment I either short, swollen, widest at apex, about twice as long as width at apex (*S.nitidus* species group, Fig. 5), or cylindrical, thin (other species, Fig. 4); segment II shorter than head width, in *S.nitidus* species group swollen along entire length, usually distinctly fusiform, in other species rod-shaped, slightly dilated distally; segments III and IV filiform; labium reaching meso- or metacoxa. ***Thorax***: Pronotum trapezoidal, about twice as broad as long, calli indistinct; mesonotum only slightly exposed; metathoracic scent gland evaporatory area broadly triangular, peritreme oval, broadly rounded apically. Metathoracic spiracle with well-developed sculpture dorsally (Fig. 6E, F). Cuneal fracture deeply incised at base. ***Legs***: Comparatively short, femora swollen, broader medially, tibia cylindrical, second and third tarsal segments of nearly equal length, claw (Fig. 6A, B) with relatively narrow base, strongly bent at midpoint, pulvillus small, not reaching or barely reaching midpoint of claw, attached to the claw along entire length; parempodia apically spatulate.

**Figure 9.**
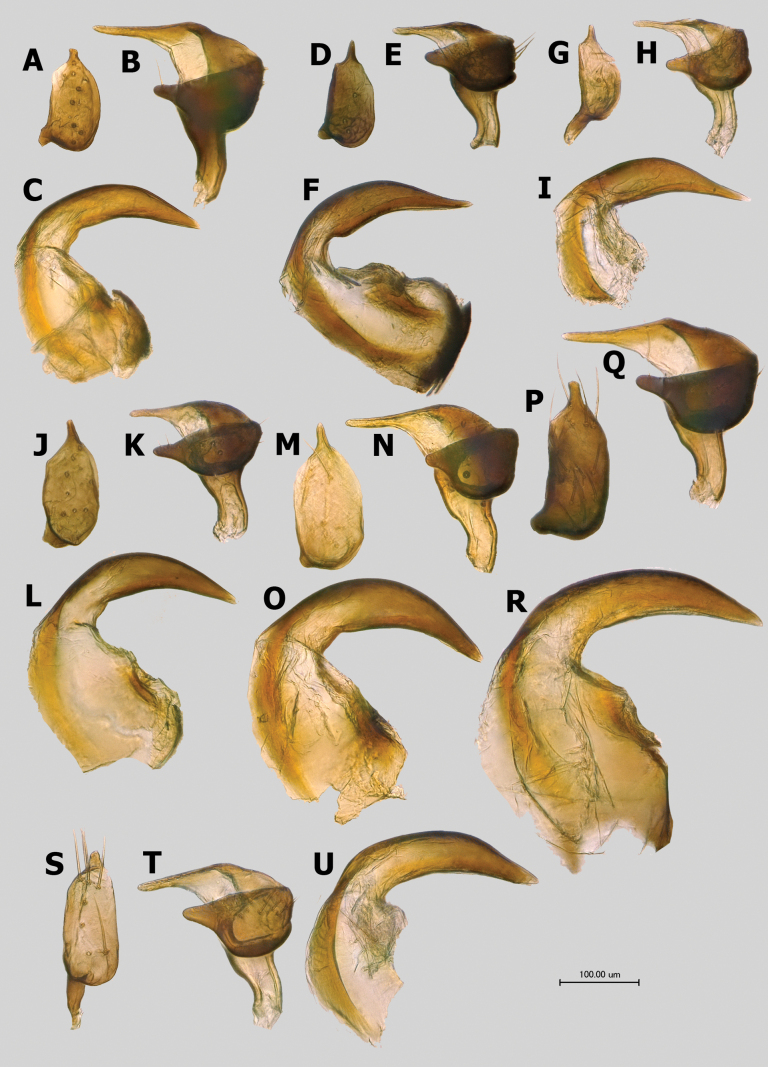
Male genitalia **A–C***Salicarusconcinnus*, paratype **D–F***S.genistae***G–I***S.nitidus***J–L***S.fulvicornis***M–O***S.halimodendri*, paratype **P–R***S.roseri***S–U***S.urnammu***A D G J M P S**: right paramere **B E H K N Q U**: left paramere **C F I L O R U**: apex of phallotheca.

***Genitalia*.** Genital capsule cone-shaped, without distinctive ornamentation, as long as or slightly longer than wide at base. Sclerotized apical part of phallotheca narrow, beak-shaped, somewhat constricted at base (Fig. 9C, F, I, L, O, R, U). Right paramere oval to elongate-oval, usually basally broadly rounded, and well expanded proximally beyond basal process, with contrastingly long, straight, apical process (Fig. 9A, D, G, J, M, P, S). Left paramere of typical phyline shape, with straight apical process and triangular, sensory lobe (Fig. 9B, E, H, K, N, Q, T). Vesica large, strongly coiled at middle, with two long and thin, gradually tapering apical blades tightly fused along most length; secondary gonopore large, located close to middle of vesica, with small gonopore sclerite (Figs 7, 8).

**Female. *Coloration, surface, and vestiture*.** As in male. ***Structure*.** Similar to male, usually smaller on average (Table 1). Antennal segment II in *S.nitidus* species group somewhat shorter and more strongly swollen, distinctly fusiform.

**Figure 10.**
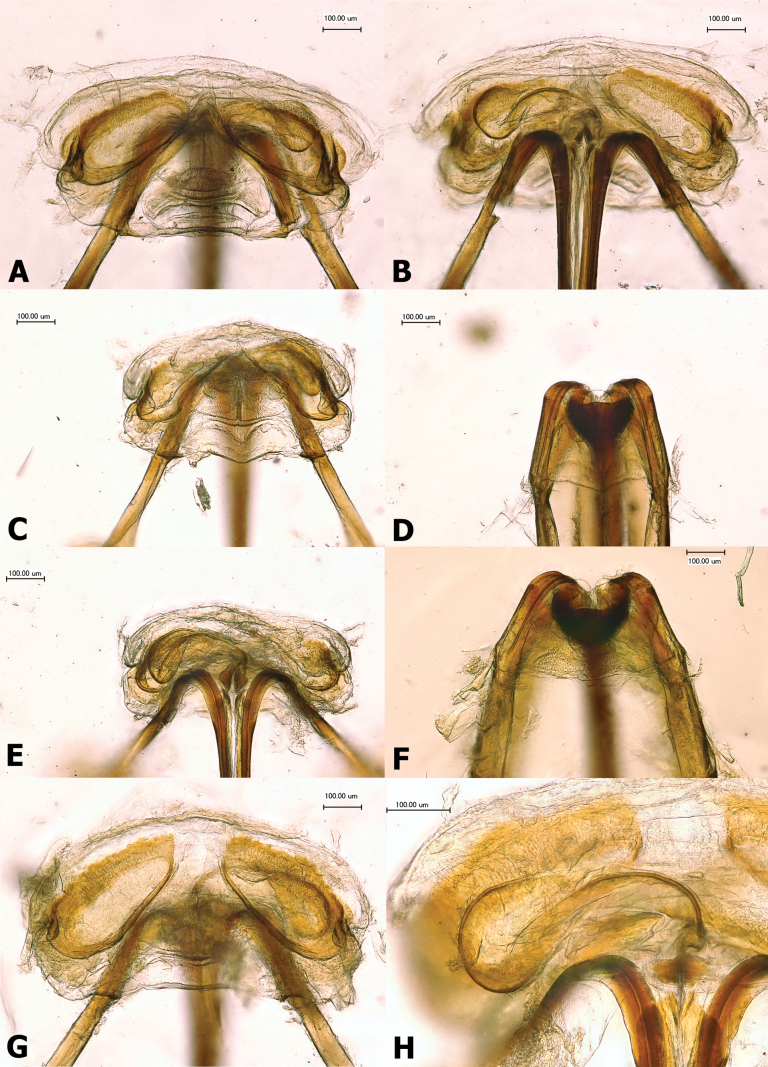
Female genitalia **A, B***Salicarusconcinnus*, paratype: **A** dorsal labiate plate **B** vestibulum **C–E***S.genistae*: **C** dorsal labiate plate **D** posterior wall **E** vestibulum **F***S.halimodendri*, posterior wall **G, H***S.roseri*: **G** dorsal labiate plate **H** vestibulum.

***Genitalia*.** Dorsal labiate plate with large and wide, broadly oval or apically tapering sclerotized rings (Fig. 10A–C, G). Posterior wall membranous, with indistinctly bordered longitudinal sclerotized bands at sides (Fig. 10D, F). Sclerites encircling vulva triangular, symmetrical (Fig. 10 E, H). Vestibulum characteristically long and thin, S-shaped (Fig. 10B, E, H).

#### Species groups.

Three distinct groups of species can be recognized within *Salicarus*, and the species treatments below are arranged alphabetically within each group:

***Salicarusnitidus* species group.** This group includes *S.cavinotum*, *S.genistae*, *S.nitidus*, and *S.perpusillus*. Species in this group are characterized by their uniformly dark color, dorsum densely covered with wide, apically serrate scale-like setae (scales type 2 sensu Stonedahl 1990), small size with a stumpy body, and total length ranging from 2.0 to 2.8. They have strongly swollen antennal segments I and II, with segment II distinctly fusiform. Species of this group have a Euro-Mediterranean distribution and utilize legumes (Fabaceae) of the tribe Genisteae (*Genista*, *Calicotome*, *Echinospartum*) as hosts.

***Salicarusroseri* species group.** This group includes *S.concinnus*, *S.roseri*, and *S.urnammu*. Species in this group are characterized by their highly variable color pattern, relatively large, oval body with a total length of 3.0–4.0. The dorsum vestiture consists of short, adpressed simple setae, while narrow, apically acuminate scale-like setae (scales type 1 sensu Stonedahl 1990) are scarce and limited to the hemelytron (if present). The vesica in these species is relatively large, with short and robust, knife-shaped apical blades. Species in this group feed on *Salix* spp. and tend to have a wide distribution: Palearctic in the case of *S.roseri*, Central Asia for *S.concinnus*, and western Asia for *S.urnammu*.

***Salicarusfulvicornis* species group.** This group includes *S.halimodendri* and *S.fulvicornis*. Species in this group are variable in coloration, with elongate males (3.6–4.0) and more ovoid females (3.5–3.9). The entire dorsum, except the head, is clothed with a mixture of silvery narrow, apically acuminate scale-like setae, and dense, comparatively long simple setae that are approximately 1.5× as long as the scales. The apical blades of the vesica are very long, thin, gradually curved, and abruptly furcate. Species of this group feed exclusively on *Caragana* spp. (Fabaceae: Hedysareae) and are mainly distributed in Central Asia and Mongolia.

### ﻿Key to species

**Table:** 

1	Smaller than 2.8. Antennal segment II distinctly swollen, fusiform. Dorsum and venter, including head, pronotum and abdomen, with dense, wide, apically serrate scales (Fig. 5)	**2**
–	Larger than 3.0. Antennal segment II thin. Scale-like setae on dorsum, if present, narrow and apically acuminate (Fig. 4)	**5**
2	Femora with scale-like setae	**3**
–	Femora without scale-like setae	**4**
3	Apical blades of vesica long, gradually curving, closely located but separate, not adjoining each other (Fig. 8D, E, I). Southern Spain, southern France, southern Italy	***S.nitidus***
–	Apical blades of vesica gradually curved and tightly adjoining each other along their entire length (Fig. 8F, G). Spain, southern France, Greece	***S.perpusillus***
4	Antennal segment II distinctly fusiform in both sexes, 1.6–1.8× as wide at midpoint as segment I at apex (Fig. 5A, B). Apical blades of vesica comparatively long, with length of larger blade distinctly exceeding distance between its base and secondary gonopore (Fig. 8A). Greece	***S.cavinotum***
–	Antennal segment II in male swollen along entire length, slightly fusiform, with midpoint width subequal to apical width of segment I (Fig. 5G). In female segment II fusiform 1.5–1.6× as wide at midpoint as segment I at apex (Fig. 5C). Apical blades of vesica comparatively short, with length of larger blade subequal to distance between its base and secondary gonopore (Fig. 8B, C). Cyprus	***S.genistae***
5	Hemelytron without scale-like setae, clothed with short, strongly adpressed, simple silvery setae only (Fig. 2F–I). Vesica with straight, short and robust, diverging apical blades (Fig. 7G, H). Widely distributed in the Palearctic. On *Salix* spp.	***S.roseri***
–	Hemelytron clothed with a mixture of simple setae and narrow, apically acuminate scale-like setae (Fig. 3C, F, I)	**6**
6	Dorsum clothed with short, subequal in length to scale-like setae on hemelytron, adpressed simple setae; narrow scale-like setae scarce and present on hemelytron only (Fig. 4C, O). Vesica with straight, short and robust apical blades (Fig. 7A, B, I, J). On *Salix* spp.	**7**
–	Pronotum, scutellum, and hemelytron clothed with a mixture of silvery narrow scale-like setae and dense, long, simple setae ~ 1.5× as long as scales (Fig. 4 D–I). Vesica with long and thin apical blades (Fig. 7C–F). On *Caragana* spp.	**8**
7	Vesica with almost parallel apical blades (Fig. 7A, B). Color-pattern of dorsum variable, ranging from entirely or largely brown to pale yellow with darkened basal margin of pronotum (Fig. 1A–F). Central Asia	***S.concinnus***
–	Vesica with gradually diverging apical blades (Fig. 7I, J). Dorsum yellow, frequently with orange tinge, sometimes with partly brown pronotum, scutellum, and endocorium (Fig. 3A–D). Southwest Asia	***S.urnammu***
8	Coloration of dorsum variable, ranging from almost entirely dark brown to pale yellow, but vertex always dirty to whitish yellow along posterior margin (Fig. 2A–D). Body in male elongate-oval, 2.7–2.9× as long as posterior width of pronotum. Apical blades of vesica abruptly furcate, with one being distinctly smaller than the other (Fig. 7E, F). Central Asia and Mongolia	***S.halimodendri***
–	Dorsum uniformly dark brown to brown (Fig. 1G–I). Body in male elongate, almost parallel-sided, 3.1–3.6× as long as width of pronotum at base. Both blades of vesica long, slightly diverging (Fig. 7C, D). Mongolia and adjacent regions of Russia and China	***S.fulvicornis***

### ﻿*Salicarusnitidus* species group

#### ﻿ Salicarus cavinotum

Taxon classificationAnimaliaHemipteraMiridae

(Wagner, 1973)

B8F10529-925F-5C60-922B-9167C31ACF51

Figs 3E, F , 5A, B , 8A

Heterocapillus cavinotum Wagner, 1973: 121.
Heterocapillus cavinotum : Wagner (1975a): 128 (key, description, figures); Linnavuori (1999): 58 (figures, updated diagnosis); Kment et al. (2005): 12 (new record).
Salicarus cavinotum : Konstantinov (2023): 861 (phylogenetic placement, figures, discussion).

##### Material examined.

***Holotype***: ♂ **Greece** • ***Dodecanese Islands***: Petaloudes, Rhodos, 36.444°N, 28.222°E, 01 Jun 1972, Eckerlein (AMNH_PBI 00184018) (ZMUH). ***Paratypes*: Greece** • ***Dodecanese Islands***: Petaloudes, Rhodos, 36.444°N, 28.222°E, 01 Jun 1972, Eckerlein, 1♂ (AMNH_PBI 00184019), 1♀ (AMNH_PBI 00336963) (ZMUH).

***Other specimens examined*: Greece** • ***Dodecanese Islands***: Petaloudes, Rhodos, 36.444°N, 28.222°E, 01 Jun 1972, Eckerlein, 1♂ (AMNH_PBI 00240965) (ZISP) • ***Peloponnese*: Corinth (Korinthia)**: nr Kehries, 37.885°N, 22.9875°E, 26 May 1989, R. Linnavuori, 2♂ (AMNH_PBI 00338309, AMNH_PBI 00338310) (NMWC) • Karitena, 37.46667°N 22.03333°E, 02 Jul 2007, A. Matocq, 7♀ (ZISP_ENT 00011853, ZISP_ENT 00011852), 4♂ (ZISP_ENT 00011853) (ZISP) • ***Thessalia***: Magnesia Co.: nr Goritsa, 39.35389°N, 22.97694°E, 03 Jun 1989, R. Linnavuori, 3♀ (ZISP_ENT 00011721), 3♂ (ZISP_ENT 00011721) (NMWC).

##### Diagnosis.

Recognized by the small size, body length 2.0–2.6; antennal segment II fusiform in both sexes, wider in female; dorsum uniformly dark brown, with dense, wide and apically serrate silvery scale-like setae (Figs 3E, F, 5A, B); legs and antennae without scales; apical blades of vesica gradually curved and tightly adjoining each other along their entire length, comparatively long, with length of larger blade distinctly exceeding distance between its base and secondary gonopore (Fig. 8A).

*Salicaruscavinotum* is most similar to *S.perpusillus* in general appearance, size, and vesica structure, which appear indistinguishable between these species (Fig. 8A, F, G). However, the latter species can be distinguished from *S.cavinotum* by the presence of dense scale-like setae on all femora, the bases of tibiae, antennal segment I, and the base of segment II (Fig. 5D–F). Additionally, in *S.perpusillus*, antennal segment II in males is 4.3–4.6× as long as wide at the midpoint and appears only slightly narrower than in females, while in *S.cavinotum* this segment in male is less fusiform, 4.9–5.3× as long as wide at the midpoint. Refer to the diagnosis of *S.genistae* for additional discussion.

##### Redescription.

**Male. *Coloration*.** Dorsum and venter uniformly brown to dark brown (Fig. 3E). ***Head***: Brown to dark brown, apices of labial segments I and II usually pale brown; antennal segments III and IV uniformly pale yellow. ***Thorax***: Uniformly brown to dark brown, extreme apices of fore and middle femora pale brown to dirty yellow, tibiae dirty yellow, with small but distinct round spots at bases of tibial spines; tarsi pale yellow, with darkened apices; membrane and veins uniformly brown. ***Abdomen***: Uniformly dark brown.

***Surface and vestiture*.** Smooth, moderately shiny; dorsum, thoracic pleura, and abdomen with dense, silvery, broad and apically serrate scale-like setae and adpressed to semierect, long, almost twice as long as scales, simple setae, dark on cuneus and apex of corium, yellowish elsewhere; legs and antennae without scale-like setae; sides of pronotum and hemelytron at base with robust, long, erect to semierect, black bristle-like setae.

***Structure*.** Body 2.4–3.0× as long as posterior width of pronotum; total length 2.0–2.6; vertex 2.3–2.7× as wide as eye; antennal segment I short, swollen, widest at apex, about twice as long as width at apex; segment II fusiform, 1.6× as wide at midpoint as segment I at apex, 4.9–5.3× as long as wide, 0.6× as long as posterior width of pronotum, 0.7–0.8× as long as width of head; segments III and IV filiform; pronotum 2.1–2.4× as wide as long, 1.2–1.3× as wide as head.

***Genitalia*.** Right paramere with oval body about twice as long as wide, basally broadly rounded and expanded well proximally beyond basal process, apical process long and straight, apically rounded. Left paramere similar to those of *S.genistae* (Fig. 9E) and *S.nitidus* (Fig. 9H), with comparatively short and straight apical process and gradually narrowing towards apex, broadly rounded sensory lobe. Apical blades of vesica gradually curved, tightly adjoining each other along their entire length, comparatively long, with length of larger blade distinctly exceeding distance between its base and secondary gonopore (Fig. 8A).

**Female. *Coloration, surface, and vestiture*.** As in male (Fig. 3F).

***Structure*.** Body 2.2–2.5× as long as posterior width of pronotum; total length 2.1–2.3; vertex 2.5–2.9× as wide as eye; antennal segment I short, swollen, widest at apex, about twice as long as width at apex; segment II fusiform, wider than in male, 1.7–1.8× as wide at midpoint as segment I at apex, 4.1–4.3× as long as wide, 0.5–0.6× as long as posterior width of pronotum, 0.7–0.8× as long as width of head; pronotum 2.1–2.4× as wide as long, 1.3× as wide as head.

***Genitalia*.** Dorsal labiate plate with large and wide, broadly oval, but apically tapering sclerotized rings.

##### Distribution.

Currently this species is documented exclusively in Greece, spanning Thessaly, the Peloponnese and Attic peninsulas, as well as Crete and Rhodes Island (Wagner 1973; Linnavuori 1999; Kment et al. 2005)

##### Hosts.

*Genista* sp. (Wagner 1975a), *Genistaacanthoclada* DC (Linnavuori 1999).

##### Discussion.

Wagner (1973, 1975a) highlighted the significance of paired rounded pits on the pronotum as the primary distinguishing feature of *S.cavinotum*, effectively distinguishing it from closely related species. Upon examination of the holotype of this species, we discovered the absence of cavities on the pronotum as described originally, albeit the designated holotype being teneral specimen with a slightly deformed pronotum, as correctly noted by Linnavuori (1999). Other specimens from the type series are in good condition and exhibit no signs of pits on pronotum (Fig. 5A, B).

#### ﻿ Salicarus genistae

Taxon classificationAnimaliaHemipteraMiridae

(Lindberg, 1948)

161D3642-48EE-5B2D-82DE-69249D86D82E

Figs 3G, H , 5C, G , 8B, C, H , 9D–F , 10C, E

Atractotomus genistae Lindberg, 1948: 53.
Heterocapillus genistae : Wagner (1975a): 126 (key, description, figures); Linnavuori (1999): 58 (figures of antennae and vesica, discussion).
Salicarus genistae : Konstantinov (2023): 861 (phylogenetic placement, figures, discussion).

##### Material examined.

***Paralectotypes*: Cyprus** • Ayios Hilarion, 35.3125°N, 33.28333°E, 07 Jun 1939, Hakan Lindberg, 1♂ (AMNH_PBI 00336958) (ZMUH) • Troodos Mesopotamos, 34.896°N, 32.908°E, 21 Jun 1939, Hakan Lindberg, 1♀ (AMNH_PBI 00336965) (ZMUH).

***Other specimens examined*: Cyprus** • Kakomallis Mt., 34.83333°N, 33.03333°E, 914 m, 13 Jun 1965, G. Mavromoustakis, 2♂ (AMNH_PBI 00336959, AMNH_PBI 00336960), 2♀ (AMNH_PBI 00336966, AMNH_PBI 00336967) (ZMUH) • Kalokhorio, 34.845°N, 33.034°E, 762 m, 29 Jun 1956, Unknown collector, 1♀ (AMNH_PBI 00240953), 2♂ (AMNH_PBI 00240954, AMNH_PBI 00240946) (ZISP) • Pano Lefkara, 34.869°N, 33.302°E, 28 May 1972, Eckerlein, 6♂ (AMNH_PBI 00240947-AMNH_PBI 00240952), 1♀ (AMNH_PBI 00240955) (ZISP).

##### Diagnosis.

Recognized by the relatively small, stumpy, dark brown body, total length male 2.2–2.5, female 2.6–2.8 (Fig. 3G, H); dorsum with dense, wide and apically serrate white scales; legs and antennae without scales; antennal segment II in male swollen, somewhat fusiform, subequal in width at midpoint to apical width of segment I, 6.4–6.7× as long as wide, in female distinctly fusiform, 1.5–1.6× as wide at midpoint as segment I at apex, 4.9–5.2× as long as wide (Fig. 5C, G); apical blades of vesica tightly adjoining each other along their entire length, comparatively short, with length of larger blade subequal to distance between its base and secondary gonopore (Fig. 8B, C, H).

*Salicarusgenistae* is most similar in habitus, coloration, size, and male genitalia structure to *S.cavinotum*, *S.nitidus*, and *S.perpusillus*. It differs habitually from these species by its sexually dimorphic antennal segment II: in males it is slightly fusiform with the width at the midpoint being subequal to the apical width of segment I; in females it is distinctly fusiform, being 1.5–1.6× as wide at the midpoint as segment I at the apex. Consequently, antennal segment II is 6.4–6.7× as long as wide in males of *S.genistae*, being 4.9–5.2× as long as wide in female. In other three closely related species this ratio ranges 4.1–5.3× in males and 3.9–4.3× in females. However, these ratios should be used with caution due to observed polymorphism and the extremely dense vestiture of antennal segment II, which can affect the measurements. *Salicarusgenistae* further differs from both *S.nitidus* and *S.perpusillus* in the absence of scales on femora. In vesica structure, with the apical blades tightly adjoining each other, it is most similar to *S.cavinotum* (Fig. 8A) and *S.perpusillus* (Fig. 8F, G), whereas in *S.nitidus* blades are apically separated. However, the vesica in *S.genistae* is slightly larger than in both *S.cavinotum* and *S.perpusillus*, and differs by having shorter apical blades, with the length of the larger blade subequal to the distance between its base and the secondary gonopore.

##### Redescription.

**Male. *Coloration*.** Dorsum and venter uniformly chestnut to dark brown (Fig. 3G). ***Head***: Brown to dark brown, buccula and apices of labial segments I and II usually paler; antennal segments III and IV uniformly pale yellow. ***Thorax***: Uniformly brown to dark brown, tibiae dirty yellow, rarely somewhat darkened basally, with small, sometimes indistinct round spots at bases of tibial spines; tarsi pale yellow, with darkened apices; membrane and veins uniformly brown. ***Abdomen***: Uniformly dark brown.

***Surface and vestiture*.** Smooth, moderately shiny; dorsum, thoracic pleura, and abdomen with dense, silvery, broad and apically serrate scale-like setae and adpressed to semierect, long, almost twice as long as scales, simple setae, dark on cuneus and extreme apex of corium and goldish yellow elsewhere (Fig. 5G); in addition, sides of pronotum and hemelytron at base with robust dark contrastingly long, erect to semierect simple setae; appendages with simple, adpressed to semierect pale setae, contrastingly dense, dark and robust on antennal segments I and II; tibial spines dark brown to black.

***Structure*.** Body stumpy, oval, 2.1–2.4× as long as posterior width of pronotum; total length 2.2–2.5; vertex flat, 2.0–2.2× as wide as eye; segment I short, swollen, widest at apex, about twice as long as width at apex; segment II swollen along entire length, somewhat fusiform, with midpoint width subequal to apical width of segment I, 6.4–6.7× as long as wide, 0.5–0.6× as long as basal width of pronotum, 0.7–0.8× as long as width of head; segments III and IV filiform; labium reaching metacoxa; pronotum 2.2–2.4× as wide as long, 1.4–1.5× as wide as head; mesonotum only slightly exposed.

***Genitalia*.** Right paramere oval, approximately twice as long as wide, basally broadly rounded, and well expanded proximally beyond basal process, with long, straight, gradually tapering apical process (Fig. 9D). Left paramere with short and straight apical process and triangular, apically broadly rounded sensory lobe (Fig. 9E). Vesica with comparatively short apical blades tightly adjoining each other along their entire length, larger blade subequal in length to distance between its base and secondary gonopore (Fig. 8B, C).

**Female. *Coloration, surface, and vestiture*.** As in male (Fig. 3H).

***Structure*.** Body 2.5–2.7× as long as posterior width of pronotum; total length 2.6–2.8; vertex 2.2–2.6× as wide as eye; antennal segment I short, swollen, widest at apex, about twice as long as width at apex; segment II somewhat shorter than in male, strongly swollen, fusiform, 1.5–1.6× as wide at midpoint as segment I at apex, 4.9–5.2× as long as wide, 0.5–0.6× as long as posterior width of pronotum, 0.7–0.8× as long as width of head; pronotum 2.1–2.2× as wide as long, 1.3–1.4× as wide as head.

***Genitalia*.** Dorsal labiate plate with large and wide, broadly oval, but apically tapering sclerotized rings (Fig. 10C). Posterior wall membranous, somewhat strongly sclerotized at sides (Fig. 10D). Vestibulum long and thin, S-shaped (Fig. 10E).

##### Distribution.

Originally described and still known from Cyprus. A record from Manavgat (Antalya province of Turkey) based on a single specimen of unknown sex (Lodos et al. 2003) requires confirmation.

##### Host.

*Genistafasselata* Decne. (Lindberg 1948, as *G.sphacelata*). An indication of *Onopordum* sp. (Asteraceae) as host (Lodos et al. 2003) certainly represents a sitting record.

##### Discussion.

Refer to the corresponding section in the redescription of *S.nitidus*.

#### ﻿ Salicarus nitidus

Taxon classificationAnimaliaHemipteraMiridae

(Horváth, 1905)

F2E88347-6B6C-5242-B374-B990C078B323

Figs 3I, G , 5H, I , 8D, E, I , 9G–I

Atractotomus nitidus Horváth, 1905: 275.
Heterocapillus nitidus : Wagner (1975a): 127 (key, description, figures); Heckmann et al. (2015): 95 (figure of vesica).
Salicarus nitidus : Konstantinov (2023): 861 (phylogenetic placement, figures, discussion).

##### Material examined.

***Holotype*: Spain** • ***Castile-La Mancha***: ♀ Pozuelo de Calatrava 38.91°N, 3.84°W, Collection date unknown, José María de la Fuente (HNHM) (not seen; pictures of the head and habitus in dorsal and lateral views were examined).

***Other specimens examined*: France** • **Corse (Corsica)**: Costa, 42.0333°N, 8.95°E, 19 Jul 1963, Unknown collector, 1♀ (AMNH_PBI 00336969) (ZMUH) • Tiuccia, 42.06566°N, 8.73889°E, 10 m, 19 Jun 1961, J. Péricart, *Calicotomevillosa* (Fabaceae), 1♂ (AMNH_PBI 00336961), 1♀ (AMNH_PBI 00336968) (ZMUH) • ***Midi-Pyrenees***: Vernet, 43.18305°N, 1.6°E, 700 m, 06 Jun 1962, J. Péricart, 1♂ (AMNH_PBI 00336964) (ZMUH).

##### Diagnosis.

Recognized by the small, stumpy, uniformly dark brown body, total length male 2.3–2.6, female 2.2–2.4; dorsum with dense, wide, and apically serrate scale-like setae, femora also clothed with scales (Fig. 3I, G); antennal segment II distinctly fusiform, at midpoint male 1.4–1.5×, female 1.6–1.7× as wide as segment I at apex (Fig. 5H, I); apical blades of vesica long, gradually curving, closely located but separate, not adjoining each other (Fig. 8D, E, I).

Most similar to *S.perpusillus* in size, body proportions, and the distinctly fusiform antennal segment II in both sexes, as well as the presence of scale-like setae on femora (although scales are absent on the bases of tibiae and antennae). However, it differs from this species, as well as from *S.cavinotum* and *S.genistae* in the vesica structure with apical blades that are not tightly adjoining. Additionally, *S.nitidus* is the only one of the closely related species mentioned above that feeds on *Calicotome* rather than *Genista* spp. Refer to the diagnosis of *S.genistae* for additional discussion.

##### Redescription.

**Male. *Coloration*.** Dorsum and venter uniformly brown to dark brown (Fig. 3I). ***Head***: Brown to dark brown, apices of labial segments I and II usually pale brown; antennal segments III and IV uniformly pale yellow. ***Thorax***: Brown to dark brown, tibiae dirty yellow, with round spots at bases of tibial spines, very small on fore and middle tibiae, distinct on hind tibia; tarsi pale yellow, with darkened apices; membrane and veins uniformly brown. ***Abdomen***: Uniformly dark brown.

***Surface and vestiture*.** Smooth, moderately shiny; dorsum, thoracic pleura, femora, and abdomen with dense, silvery, broad and apically serrate scale-like setae and adpressed to semierect, long, almost twice as long as scales, simple setae, dark on cuneus and apex of corium, yellowish elsewhere; series of long simple setae on fore coxa silver; sides of pronotum and hemelytron at base with robust, long, erect to semierect, black bristle-like setae.

***Structure*.** Body 2.5–2.7× as long as posterior width of pronotum; total length 2.3–2.6; vertex 2.3–2.6× as wide as eye; antennal segment II distinctly fusiform, 1.4–1.5× as wide at midpoint as segment I at apex, 4.1–4.3× as long as wide, 0.5–0.6× as long as posterior width of pronotum, 0.7× as long as width of head; pronotum 2.1–2.3× as wide as long, 1.3× as wide as head.

***Genitalia*.** Right paramere spoon-shaped, with long, straight, and blunt apical process (Fig. 9G). Right paramere with thin, short, and straight apical process and broadly rounded sensory lobe (Fig. 9H). Vesica with very long, gradually curving, apical blades, closely located but separate, not adjoining each other (Fig. 8D, E, I).

**Female. *Coloration, surface, and vestiture*.** As in male (Fig. 3G).

***Structure*.** Body 2.3–2.5× as long as posterior width of pronotum; total length 2.2–2.4; vertex 2.4–2.6× as wide as eye; antennal segment II somewhat wider than in male, 1.6–1.7× as wide at midpoint as segment I at apex, 4.0–4.1× as long as wide, 0.6× as long as posterior width of pronotum, 0.7× as long as width of head; pronotum 2.2–2.3× as wide as long, 1.3–1.4× as wide as head.

***Genitalia*.** Dorsal labiate plate with large and wide, broadly oval at base, apically tapering sclerotized rings.

##### Distribution.

Ciudad Real province of Spain (Wagner 1975a: Pozuelo de Calatrava), southern France (Wagner 1975a: Corsica), southern Italy (Carapezza 1984: Sardinia; Carapezza 1993: Aeolian Islands).

##### Host.

*Calicotomevillosa* (Poir.) Link. (Wagner 1975a). *Genistacorsica* (Loisel.) DC was reported as a host from Sardinia (Carapezza 1984) and *Genistaephedroides* DC. from Aeolian Islands (Carapezza 1993). However, the last two records may pertain to *S.perpusillus* and require further confirmation (Carapezza 1995).

##### Discussion.

*Salicarusnitidus* (Horváth, 1905) belongs to a group of four hardly distinguishable species with a complex taxonomic history, which also includes *S.cavinotum* (Wagner, 1973), *S.genistae* (Lindberg, 1948), and *S.perpusillus* (Wagner, 1960). These species inhabit the North Mediterranean, from central Spain in the West to Cyprus in the East. Horváth (1905) described *Atractotomusnitidus* based on a single female from central Spain and noted its similarity with *Atractotomus* (currently *Heterocapillus*) *tigripes* (Mulsant & Rey, 1852) due to its overall appearance and coloration, as well as the presence of large dark spots at bases of tibial spines. He distinguished *A.nitidus* by its much smaller size (2.25 mm), less fusiform antennal segment II, and tibiae not darkened ventrally.

Lindberg (1948) described *A.genistae* from a series of specimens collected in two localities in Cyprus. He emphasized the similarity of the new species to *Atractotomusmali* (Meyer-Dur, 1843) due to the spindle-shaped antennal segment II in both sexes. According to the original description, the new species differs in having a dark-colored membrane, dark spots at the bases of tibial spines, smaller size, and a shorter segment II, which is distinctly shorter than the head width. Lindberg (1948) did not mention *A.nitidus* in his diagnosis and was apparently unaware of this species.

Wagner (1960) described *Atractotomusperpusillus* from Sierra Nevada (southern Spain) as the smallest species of the genus (2.1–2.4 mm), most similar to *A.parvulus* Reuter, 1878, but differing from that species in having wide scale-like setae, a spindle-shaped antennal segment II, and a long and thin apical process of the vesica. Wagner also noted that the dorsal vestiture of *A.perpusillus*, with three types of setae, separates it from all congeners except *A.tigripes*. To accommodate these two species and *A.putoni* Reuter, 1878 (subsequently synonymized with *A.validicornis* Reuter, 1876), he erected the subgenusHeterocapillus Wagner, 1960 which was later upgraded to a valid genus by Kerzhner (1962). The last species of this group, *Heterocapilluscavinotum*, was described by Wagner (1973) from Rhodos Island (Greece). According to the original description, this species is most similar to *H.nitidus* and *H.perpusillus* but differs from both in having a pair of rounded pits on the pronotum, and a set of minor distinctions some of which, such as body length, appear to contradict with provided measurements.

In his monographic treatment of Mediterranean plant bugs Wagner (1975a) formulated the species concepts of these four closely related species as follows:

*Heterocapillusgenistae* (Cyprus, on *Genista* sp.): relatively large, body length male 2.5 mm, female 3.0 mm, antennal segment II slightly spindle-shaped in male, distinctly spindle-shaped in female.

*Heterocapilluscavinotum* (Rhodos Island, on *Genista* spp.): body length male 2.35 mm, female 1.9 mm, pronotum with a pair of pits, antennal segment II distinctly spindle-shaped in both sexes, 3.8–4.0× as long as wide at middle.

*Heterocapillusnitidus* (central Spain, Corsica, on *Calicotomevillosa*): body length male 2.5 mm, female 2.2–2.3 mm, antennal segment II spindle-shaped in both sexes, male 4×, female 3.9× as long as wide at middle.

*Heterocapillusperpusillus* (southern Spain, southern France, on *Genista* sp.): body length male 2.1 mm, female 2.3–2.4 mm, antennal segment II spindle-shaped in both sexes, male 4.2×, female 4.0× as long as wide at middle.

Since then, *H.cavinotum* was additionally reported from Peloponnese peninsula and Crete (Linnavuori 1999; Kment et al. 2005), while the presence of pits on the pronotum in this species was refuted (Linnavuori 1999). *Heterocapillusnitidus* was additionally indicated from Sardinia and Aeolian Islands (Italy, Carapezza 1984, 1993), while *H.perpusillus* from Laconia (Greece, Rieger 2012) and Crete (Heckmann et al. 2015). A synonymy of *H.perpusillus* with *H.nitidus* was suspected by Goula and Ribes (1995), but Heckmann et al. (2015) noted slight distinctions in the size and shape of the vesica between these two species. Konstantinov (2023) transferred all these species to *Salicarus*. Examination of all available material allows us to conclude that despite the notable similarity, all four species could be distinguished from each other by the combination of characters provided in the key to species and diagnoses. Molecular data are desirable for elucidating the status of these species. Pending such a study, we refrain from nomenclatorial changes.

#### ﻿ Salicarus perpusillus

Taxon classificationAnimaliaHemipteraMiridae

(Wagner, 1960)

FA72537D-88D9-584D-86B5-91BB74EE4713

Figs 3K, L , 5 D–F , 8F, G

Atractotomus (Heterocapillus) perpusillus Wagner, 1960: 81.
Heterocapillus perpusillus : Wagner (1975a): 128 (key, description, figures); Heckmann et al. (2015): 95 (figures of dorsal habitus and vesica).
Salicarus perpusillus : Konstantinov (2023): 861 (phylogenetic placement, figures, discussion).

##### Material examined.

***Holotype*: Spain** • ***Andalucia***: ♂ Northern Slopes of Veleta Peak [Veleta -Nordhang], Sierra Nevada, 37.07°N, 3.37°W, 2500 m, 03 Aug 1959, E. Wagner, (AMNH_PBI 00184020) (ZMUH). ***Paratypes*: Spain** • ***Andalucia***: Northern Slopes of Veleta Peak [Veleta -Nordhang], Sierra Nevada, 37.07°N, 3.37°W, 2500 m, 02 Aug 1959, E. Wagner, 1♀ (AMNH_PBI 00336976) (ZMUH); 03 Aug 1959, E. Wagner, 1♂ (AMNH_PBI 00336979), 3♀ (AMNH_PBI 00336974, AMNH_PBI 00336975, AMNH_PBI 00336978) (ZMUH) • Sierra Nevada Veleta, 37.08333°N, 3.16667°W, 25 Jul 1959–04 Aug 1959, H. H. Weber, 2♀ (AMNH_PBI 00126474, AMNH_PBI 00126475) (ZSMC).

***Other specimens examined*: Spain** • ***Catalonia***: Campllong, Bergueda, 41.88333°N, 2.81667°E, 15 Jul 1984, E. Ribes, 1♂ (ZISP_ENT 00011719), 1♀ (ZISP_ENT 00011719) (NMPC) • Seros, Segria, 41.462°N, 0.412°E, 27 Jun 1971, J. Ribes, *Ulex* sp. (Fabaceae), 1♂ (AMNH_PBI 00338308) (NMWC) • Sonadell, Lleida, 02 Jun 1963, J. Ribes, 1♂ (AMNH_PBI 00338307) (NMWC).

##### Diagnosis.

Recognized by the small and stumpy body, total length 2.1–2.4; dorsum uniformly dark brown with dense, wide and apically serrate scale-like setae (Fig. 3K); femora, bases of tibiae, segment I and base of segment II with also covered with wide silvery scales (Fig. 5F); antennal segment II distinctly fusiform, at middle male 1.3–1.5×, female 1.6–1.7× as wide as segment I at apex (Fig. 5D, E); apical blades of vesica gradually curved and tightly adjoining each other along their entire length, comparatively long, with length of larger blade distinctly exceeding distance between its base and secondary gonopore (Fig. 8F, G).

*Salicarusperpusillus* is habitually most similar to *S.nitidus* in body proportions, the distinctly fusiform antennal segment II in both sexes, and the presence of scale-like setae on the femora. However, the latter species differs from *S.perpusillus* in its distinctive vesica structure with separate, not tightly adjoining apical blades. Indistinguishable from *S.cavinotum* in vesica structure but differs from that species by the presence of dense scale-like setae on the femora.

##### Redescription.

**Male. *Coloration*.** Dorsum and venter uniformly brown to dark brown (Fig. 3K). ***Head***: Brown to dark brown, apices of labial segments I and II usually pale brown; antennal segments III and IV uniformly pale yellow. ***Thorax***: Uniformly brown to dark brown, tibiae dirty yellow, with small round spots at bases of tibial spines, less distinct on fore and middle tibiae; tarsi pale yellow, with darkened segment III; membrane and veins uniformly brown. ***Abdomen***: Uniformly dark brown.

***Surface and vestiture*.** Smooth, moderately shiny; dorsum, thoracic pleura, and abdomen with very dense, silvery, broad and apically serrate scale-like setae and adpressed to semierect, long, almost twice as long as scales, simple setae, dark on cuneus and apex of corium, yellowish elsewhere; antennal segments I and II, all femora and basal parts of tibiae clothed with scale-like setae; sides of pronotum and hemelytron at base with robust, long, erect to semierect, black bristle-like setae.

***Structure*.** Body 2.1–2.3× as long as posterior width of pronotum; total length 2.1–2.4; vertex 2.2–2.5× as wide as eye; segment II distinctly fusiform, 1.3–1.5× as wide at midpoint as segment I at apex, 4.3–4.6× as long as wide, 0.5–0.6× as long as posterior width of pronotum, 0.7–0.8× as long as width of head; pronotum 2.1–2.2× as wide as long, 1.2–1.3× as wide as head.

***Genitalia*.** Right paramere spoon-shaped, with long, straight apical process. Right paramere with short and straight apical process and broadly rounded sensory lobe. Vesica with gradually curved and comparatively long apical blades tightly adjoining each other along their entire length, with length of larger blade distinctly exceeding distance between its base and secondary gonopore (Fig. 8F, G).

**Female. *Coloration, surface, and vestiture*.** As in male (Fig. 3L).

***Structure*.** Body 2.4–2.6× as long as posterior width of pronotum; total length 2.2–2.4; vertex 2.3–2.7× as wide as eye; segment II distinctly somewhat wider than in male, 1.6–1.7× as wide at midpoint as segment I at apex, 3.9–4.2× as long as wide, 0.6× as long as posterior width of pronotum, 0.7–0.8× as long as width of head; pronotum 2.1–2.2× as wide as long, 1.3× as wide as head.

***Genitalia*.** Dorsal labiate plate with large and wide, broadly oval at base, apically tapering sclerotized rings.

##### Distribution.

Spain (Wagner 1960: Sierra Nevada; Ehanno 1987: Navarre; Ribes et al. 2004: Catalonia; Pagola-Carte and Zabalegui 2007: Araba and Navarre), southern France (Wagner 1975a: Corsica; Ehanno 1987: Pyrénées-Orientales), Greece (Rieger 2012: Laconia; Heckmann et al. 2015: Peloponnese, Western Thrace, Crete). An indication from Italy (Faraci and Rizzotti Vlach 1995) was based on specimens collected by A. Melber in Saltino and Montemignaio, Tuscany from *Cytisusscoparius* L. (Melber 1993) and partly retained at the Museum of Verona. Franco Faraci kindly provided us with pictures of one specimen from Secchieta Mt. which may belong to *S.genistae* and requires further confirmation of the species identity.

##### Host.

*Genistaversicolor* Boiss. (Wagner 1975a, as *Genistabaetica* Spach.), *Genistascorpius* (L.) DC. (Ribes et al. 2004; Pagola-Carte and Zabalegui 2007), *Echinospartumhorridum* (Vahl) Rothm. (Ehanno 1987, as *Genistahorrida*).

##### Discussion.

Goula and Ribes (1995), followed by Kerzhner and Josifov (1999) suspected that *S.perpusillus* is a junior synonym of *S.nitidus*. Heckmann et al. (2015) argued that these species can be distinguished based on their sizes, antennal proportions, and the shape of the vesica. Our observations indicate that distinctions in size and antennal segment II are not reliable diagnostic features, but these species can be differentiated by distinctions in the mutual arrangement of their apical blades (see Diagnosis).

### ﻿*Salicarusroseri* species group

#### ﻿ Salicarus concinnus

Taxon classificationAnimaliaHemipteraMiridae

V. G. Putshkov, 1977

1F766A8B-54B8-54ED-B083-BD5C21854069

Figs 1A–F , 4A–C , 7A, B , 9 A–C , 10A, B

Salicarus (Salicarus) concinnus V. G. Putshkov, 1977: 365.
Salicarus concinnus : Konstantinov (2023): 874 (phylogenetic placement, figures, discussion).

##### Material examined.

***Holotype*: Tajikistan** • ♂ Kondara Canyon, Valley of Varzob River, 38.83333°N, 68.83333°E, 1100 m, 08 Jul 1955, Lopatin, (AMNH_PBI 00233863) (ZISP). ***Paratypes*: Kazakhstan** • ***South Kazakhstan Prov.***: Daubaba nr Tyul’kubas, Shimkent Dist., 42.46666°N, 70.26666°E, 18 Jun 1966, Unknown collector, Salix sp. (Salicaceae), 1♂ (AMNH_PBI 00233874) (ZISP). **Kyrgyzstan** • Gava, 41.26666°N, 72.83333°E, 03 Aug 1937, A. N. Kiritshenko, 2♂ (AMNH_PBI 00233872, AMNH_PBI 00233873) (ZISP). Tajikistan • Kondara Canyon, Valley of Varzob River, 38.83333°N, 68.83333°E, 1100 m, 19 Jun 1937, Gussakovskiy, 1♀ (AMNH_PBI 00233768) (ZISP) • 30 Jun 1943, A. N. Kiritshenko, 2♂ (AMNH_PBI 00233866, AMNH_PBI 00233867), 4♀ (AMNH_PBI 00233761-AMNH_PBI 00233764) (ZISP) • 05 Jul 1943, A. N. Kiritshenko, 1♀ (AMNH_PBI 00233760) (ZISP) • 10 Jun 1955, Zakieva, 1♀ (AMNH_PBI 00233769) (ZISP) • 16 Jun 1955, Lopatin, 1♀ (AMNH_PBI 00233758) (ZISP) • 08 Jul 1955, Lopatin, 1♂ (AMNH_PBI 00233864), 2♀ (AMNH_PBI 00233756, AMNH_PBI 00233757) (ZISP) • 09 Jul 1955, Lopatin, 1♂ (AMNH_PBI 00233865), 1♀ (AMNH_PBI 00233759) (ZISP) • 13 Jun 1956, Denisova and Ivanova, 3♀ (AMNH_PBI 00233765-AMNH_PBI 00233767) (ZISP) • 28 Jun 1956, Kiriyanova, 2♀ (AMNH_PBI 00233770, AMNH_PBI 00233771) (ZISP). **Uzbekistan** • Angren River, 15 km NO Angren, 41.1°N, 70.3°E, 18 Jun 1966, I. M. Kerzhner, 4♀ (AMNH_PBI 00233868-AMNH_PBI 00233871), 8♂ (AMNH_PBI 00233868-AMNH_PBI 00233871) (ZISP) • Karzhantau Mt. Ridge, 41.73333°N, 70.03333°E, 01 Jul 1939, Obukhova, *Salixwilhelmsiana* (Salicaceae), 1♀ (AMNH_PBI 00233773) (ZISP) • Tugay Ugama, Karzhantau Mt. Ridge, 41.73333°N, 70.03333°E, 17 Jul 1939, Obukhova, *Salix* sp. (Salicaceae), 4♂ (AMNH_PBI 00233875-AMNH_PBI 00233878), 1♀ (AMNH_PBI 00233772) (ZISP).

***Other specimens examined*: Tajikistan** • 6 km W Kuibyshevsk, Valley of Vakhsh River, 37.96666°N, 68.75°E, 14 Jul 1943, A. N. Kiritshenko, 1♀ (AMNH_PBI 00233774) (ZISP). **Turkmenistan** • Charshanga, 30 km W Kelif, 37.5°N, 66.015°E, 07 Jun 1934, Bregetova, 2♀ (AMNH_PBI 00233775, AMNH_PBI 00233776) (ZISP). **Uzbekistan** • Karzhantau Mt. Ridge, 41.73333°N, 70.03333°E, 01 Jul 1939, Obukhova, *Salixwilhelmsiana* (Salicaceae), 1♂ (AMNH_PBI 00233879) (ZISP).

##### Diagnosis.

Recognized by the following combination of characters: Body oval, total length 3.0–3.7; antenna uniformly pale yellow, with thin segment II (Fig. 4A, B), coloration of dorsum variable, ranging from entirely or largely brown to pale yellow with darkened basal margin of pronotum (Fig. 1A–F); hemelytron clothed with a mixture of short adpressed simple setae and scarce, narrow, apically acuminate, silvery scale-like setae (Fig. 4C); pronotum and scutellum with short simple setae only; apical blades of vesica robust, almost straight, and parallel each other (Fig. 7A, B).

*Salicarusconcinnus* is most similar in size, body proportions, vestiture, and vesica structure to *S.roseri* and *S.urnammu*. The vestiture of the dorsum in all three species is mainly composed of short, adpressed simple setae, with the addition of scarce, narrow, apically acuminate scale-like setae on the hemelytron in the case of *S.concinnus* and *S.urnammu*. In *S.roseri*, scale-like setae are present on the thoracic pleura only. The color pattern of the dorsum in these species is highly variable, although in *S.concinnus*, it tends to be more uniform, frequently being either dark brown with a yellowish vertex or whitish yellow with a darkened posterior margin of the pronotum. In contrast, in dark specimens of *S.urnammu* and pale specimens of *S.roseri*, the anterior part of the pronotum is most frequently darkened, and the hemelytron usually has a more or less darkened endocorium (Figs 2F–I, 3A–D). The vesica in these species is relatively large, with short and robust, knife-shaped apical blades. However, in both *S.roseri* and *S.urnammu*, the apical blades of the vesica are apically diverging (Fig. 7G–J), while they are parallel to each other in *S.concinnus* (Fig. 7A, B).

##### Redescription.

**Male. *Coloration*.** Variable, ranging from entirely or largely brown to pale yellow with darkened basal margin of pronotum (Fig. 1A–C). ***Head***: Brown, with narrow whitish edging along eyes gradually expanding towards vertex to whitish yellow, with large brown spot on frons, sometimes uniformly whitish yellow; vertex always whitish entirely or along posterior margin; antenna uniformly pale yellow; labrum dirty yellow; entire labium brown even in pale specimens, with dark brown segment IV. ***Thorax***: Pronotum dorsally ranging from brown, darker towards base, to pale yellow, with narrowly brown posterior margin; lateral sides of pronotum uniformly brown to pale yellow with narrow brown edging; exposed part of mesonotum and scutellum from uniformly brown to pale yellow, sides of mesonotum sometimes with orange tinge. Hemelytron uniformly brown, pale brown or whitish yellow; membrane pale brown, semitransparent. Coxae entirely or basally brown; femora always brown in basal two-thirds, with pale yellow apices; tibiae and tarsi uniformly pale yellow. Thoracic pleura always brown to dark brown. ***Abdomen***: Uniformly brown to dark brown.

***Surface and vestiture*.** Dorsum smooth, shiny. Pronotum, scutellum, and hemelytron with short, subequal in length to scale-like setae on hemelytron, adpressed simple setae, usually dark brown, sometimes yellowish; hemelytron additionally with silver scale-like setae; thoracic pleurites clothed with dense scale-like setae only (Fig. 4A–C); appendages and abdomen with thin and short, adpressed, whitish simple setae; tibial spines black.

***Structure*.** Body oval, 2.4–2.8× as long as posterior width of pronotum, total length 3.0–3.7; head vertical, rather vide, slightly protruding beyond eyes anteriorly and ventrally; vertex flat, posteriorly attenuate and covering anterior margin of pronotum, 2.1–2.3× as wide as eye; frons weakly convex; clypeus flat, not visible in dorsal view; antennal segment II thin and short, 0.5–0.6× as long as posterior width of pronotum, 0.9× as long as width of head; pronotum with broadly rounded anterior and posterior corners, 1.9–2.1× as wide as long, 1.5–1.6× as wide as head.

***Genitalia*.** Right paramere oval, ~ 1.9× as long as wide, with basal part broadly rounded and expanded proximally beyond basal process; apical process comparatively short, subrectangular (Fig. 9A). Left paramere with long, thin, and straight apical process and relatively thin, apically broadly rounded sensory lobe (Fig. 9B). Vesica relatively large, with almost straight, robust and parallel subapical blades (Fig. 7A, B).

**Female. *Coloration, surface and vestiture*.** As in male (Fig. 1D–F). ***Structure*.** Similar to male, body 2.3–2.6× as long as posterior width of pronotum; total length 3.0–3.5; vertex 2.3–2.6× as wide as eye; antennal segment II 0.5× as long as posterior width of pronotum, 0.8–0.9× as long as width of head; pronotum 2.0–2.2× as wide as long, 1.5–1.7× as wide as head.

***Genitalia*.** Dorsal labiate plate with large and broadly oval sclerotized rings (Fig. 10A). Vestibulum S-shaped, thin (Fig. 10B).

##### Distribution.

Central Asia. Known from Southern Kazakhstan, Kyrgyzstan, Tajikistan, Turkmenistan, and Uzbekistan (Putshkov 1977).

##### Hosts.

Feeds on fructiferous *Salix* spp. (Putshkov 1977).

#### ﻿ Salicarus roseri

Taxon classificationAnimaliaHemipteraMiridae

(Herrich-Schaeffer, 1838)

59892C7C-3A4A-59F5-8040-0B2F748EB95A

Figs 2F–I , 4J–L , 6D–F , 7G, H , 9D–F , 10G, H

Capsus roseri Herrich-Schaeffer, 1838: 78.
Capsus geniculatus Stål, 1858: 355 (synonymized by Thomson 1871: 449).
Capsus saliceticola Stål, 1858: 355 (synonymized by Thomson 1871: 449).
Sthenarus vittatus Fieber, 1858: 339 (synonymized by Puton 1875: 44).
Sthenarus roseri : Fieber (1861): 309 (description); Reuter (1878): 47 (description, key); Southwood and Leston (1959): 233 (description, key).
Plagiognathus (Sthenarus) roseri : Reuter (1875): 178 (description).
Lygus roseri : Vollenhoven (1875): 93 (description).
Sthenarus (Phoenicocoris) roseri : Wagner (1958): 412 (description, figures); Wagner and Weber (1964): 437 (description, figures, key).
Salicarus roseri : Kerzhner (1962): 381 (new combination, description, figures); Kerzhner (1964): 996 (key, figures); Schwartz and Stonedahl (2004): 12 (discussion, figures, SEM); Konstantinov (2023): 874 (figures, discussion).
Sthenarus (Salicarius (sic!)) roseri: Wagner (1975a): 101 (description, figures, key).

##### Material examined.

**Belarus** • Korolevo nr Vitebsk, 55.13333°N, 30.5°E, 08 Jul 1905, Birulya, 1♂ (AMNH_PBI 00233591) (ZISP). **Bulgaria** • Srouma River Valley, Topolnitsa Vill., 41.41607°N, 23.31772°E, 07 Jun 2014, Simov N., 1♂ (AMNH_PBI 00341036) (ZISP). **Georgia** • Benara, 19 km W Akhaltsykhe, 41.65°N, 42.815°E, 10 Jun 1949, A. N. Kiritshenko, *Salix* sp. (Salicaceae), 1♂ (AMNH_PBI 00233544) (ZISP) • 14 Jun 1949, A. N. Kiritshenko, 1♂ (AMNH_PBI 00233545), 1♀ (AMNH_PBI 00233472) (ZISP) • 17 Jun 1949, A. N. Kiritshenko, 4♂ (AMNH_PBI 00233546-AMNH_PBI 00233549), 4♀ (AMNH_PBI 00233473-AMNH_PBI 00233476) (ZISP) • 18 Jun 1949, A. N. Kiritshenko, 3♀ (AMNH_PBI 00233477-AMNH_PBI 00233479), 4♂ (AMNH_PBI 00233550-AMNH_PBI 00233553) (ZISP) • 19 Jun 1949, A. N. Kiritshenko, 3♂ (AMNH_PBI 00233554-AMNH_PBI 00233556), 6♀ (AMNH_PBI 00233480-AMNH_PBI 00233485) (ZISP) • 20 Jun 1949, A. N. Kiritshenko, 6♀ (AMNH_PBI 00233486-AMNH_PBI 00233491), 11♂ (AMNH_PBI 00233557-AMNH_PBI 00233567) (ZISP) • 22 Jun 1949, A. N. Kiritshenko, 1♂ (AMNH_PBI 00233568), 1♀ (AMNH_PBI 00233492) (ZISP) • 23 Jun 1949, A. N. Kiritshenko, 4♀ (AMNH_PBI 00233493-AMNH_PBI 00233496), 3♂ (AMNH_PBI 00233569-AMNH_PBI 00233571) (ZISP) • 25 Jun 1949, A. N. Kiritshenko, 3♀ (AMNH_PBI 00233497-AMNH_PBI 00233499) (ZISP) • Borzhomi [Borzhom] Tiflis Dist., 41.83333°N, 43.36666°E, 1867, A. Brandt, 1♀ (AMNH_PBI 00233913) (ZISP). **Mongolia** • ***Central Aimak***: Toola river, between Gachurin and Khuantey, NE of Ulaanbaator [Urga], 47.933°N, 107.165°E, 04 Jul 1897, Klements, 1♀ (AMNH_PBI 00233916) (ZISP) • ***South Govi Aimak***: Nr Muna-Ula Mt., Jun 1871, Przhevalskiy, 2♀ (AMNH_PBI 00233918, AMNH_PBI 00233919) (ZISP) • ***South Hangay Aimak***: Lamyn-gegen, SE Khangay, 22 Jul 1926, A. N. Kiritshenko, 1♀ (AMNH_PBI 00233928) (ZISP). **Poland** • Khabirov, Kalish Dist., 51.75°N, 18.08333°E, 19 Jun 1908, Yachevskiy, 1♂ (AMNH_PBI 00233837), 1♀ (AMNH_PBI 00233990) (ZISP) • 31 Jul 1908, Yachevskiy, 2♂ (AMNH_PBI 00233835, AMNH_PBI 00233836) (ZISP). **Russian Federation** • ***Altai Terr.***: Tigirekskiy National Reserve, 51.05°N, 82.98333°E, 26 Jun 2005, A. Namyatova, 2♀ (AMNH_PBI 00233941, AMNH_PBI 00233942) (ZISP) • 03 Jul 2005, A. Namyatova, 1♂ (AMNH_PBI 00233823) (ZISP) • ***Amur Prov.***: Klimoutsy, 40 km W of Svobodnyi, 51.4667°N, 127.5833°E, 242 m, 13 Jul 1959, I. M. Kerzhner, 1♂ (AMNH_PBI 00233931) *Salix* spp. (Salicaceae), 1♂ (AMNH_PBI 00233932) (ZISP) • 14 Jul 1959, I. M. Kerzhner, *Salix* spp. (Salicaceae), 1♂ (AMNH_PBI 00233816) (ZISP) • 15 Jul 1959, I. M. Kerzhner, 1♂ (AMNH_PBI 00233933) (ZISP) • Korsakovo on Amur River, 51.33333°N, 126.95°E, 25 Jul 1959, I. M. Kerzhner, 1♂ (AMNH_PBI 00233934) (ZISP) • ***Arkhangelsk Prov.***: Left bank of Severnaya Dvina River, oppos. Kotlas, 61.25°N, 46.51666°E, 06 Aug 1942, Stark, 1♂ (AMNH_PBI 00233818) (ZISP) • 14 Aug 1942, Stark, 1♀ (AMNH_PBI 00233937) (ZISP) • ***Chelyabinsk Prov.***: Tract nr Troitsk Region, Magnitogorsk Distr., 54.108°N, 61.568°E, 07 Jun 1927, Shelud’ko, 1♀ (AMNH_PBI 00233917) (ZISP) • ***Irkutsk Prov.***: Irkutsk, 52.31666°N, 104.23333°E, Yakovlev, 3♀ (AMNH_PBI 00233962, AMNH_PBI 00233513, AMNH_PBI 00233514), 1♂ (AMNH_PBI 00233578) (ZISP) • 18 Jul 1961, Kulik, *Salix* sp. (Salicaceae), 20♀ (AMNH_PBI 00233969-AMNH_PBI 00233972, AMNH_PBI 00233510-AMNH_PBI 00233512), 7♂ (AMNH_PBI 00233575-AMNH_PBI 00233577) (ZISP) • Padun on Verkhnyaya Tunguska River, 56.28333°N, 101.71667°E, 1867, A. Czekanowski, 1♀ (AMNH_PBI 00233924) (ZISP) • ***Kamchatka Terr.***: Klyuchevskoe on the Kamchatka River, 56.3°N, 160.83333°E, 09 Jul 1908, Bianchi, 1♀ (AMNH_PBI 00233910) (ZISP) • ***Khabarovsk Terr.***: Troitskoe, bank of Amur river, Primor’e, 49.43333°N, 136.55°E, 10 Jun 1909, Efremov, 1♀ (AMNH_PBI 00233940) (ZISP) • ***Krasnodar Terr.***: Slavyansk-na-Kubani [Slavyanskaya], lower course of Kuban river, 45.23333°N, 38.11666°E, 12 Jul 1937, Rysakov, 1♀ (AMNH_PBI 00233979), 1♂ (AMNH_PBI 00233819) (ZISP) • Tuapse, 44.1°N, 39.08333°E, 13 Jul 1911, W. Pliginskiy, 1♂ (AMNH_PBI 00233822) (ZISP) • ***Krasnoyarsk Terr.***: Krasnoyarsk, 56.00972°N, 92.79167°E, 03 Aug 1924, Vinogradov, 1♀ (AMNH_PBI 00233912) (ZISP) • ***Leningrad Prov.***: Bank of Tosna River nr Sablino, 59.62°N, 30.81°E, 12 Aug 1922, A. N. Kiritshenko, 1♂ (AMNH_PBI 00233814) (ZISP) • Bolshie Izhory [Bol’shiye Izori], 58.7986°N, 30.0786°E, 57 m, 16 Jun 1917, Bianchi, 1♀ (AMNH_PBI 00233980), 1♂ (AMNH_PBI 00233593) (ZISP) • Ivanovskoe on Neva River, 59.75°N, 30.76666°E, 20 Jul 1931, Lyubishchev, 1♀ (AMNH_PBI 00233976) (ZISP) • Novyy Petergof [Petrodvorets], 59.86666°N, 29.91666°E, 09 Jul 1896, Chekini, 1♂ (AMNH_PBI 00233587) (ZISP) • Shuvalovo, 60.05°N, 30.3°E, 25 May 1897, Jakobson, 1♀ (AMNH_PBI 00233921) (ZISP) • 05 Jun 1897–08 Jun 1897, Jakobson, 1♀ (AMNH_PBI 00233922) (ZISP) • 04 Jul 1897, Jakobson, 1♀ (AMNH_PBI 00233961) (ZISP) • St.-Petersburg [Petrograd, Leningrad], 59.935°N, 30.31°E, 1869, Solskiy, 8♀ (AMNH_PBI 00233964, AMNH_PBI 00233966, AMNH_PBI 00233590) (ZISP) • Udel’naya, 60.016°N, 30.318°E, 20 Jun 1916, Knyazhetskiy, 1♀ (AMNH_PBI 00233923) (ZISP) • ***Moscow Prov.***: Belye Kolodezi, Kolomna distr., 54.91889°N, 38.69139°E, 08 Jun 1903, G.A. Kozhevnikov, 1♀ (AMNH_PBI 00233963) (ZISP) • ***Nizhegorod Prov.***: Pamyati Parizhskoi Kommuny inlet, 56.099°N, 44.516°E, 12 Jul 1979, Khrynova, 1♂ (AMNH_PBI 00233820) (ZISP) • ***North Ossetia Rep.***: Vladikavkaz, 43.01666°N, 44.66666°E, 11 Jul 1925, A. N. Kiritshenko, 1♀ (AMNH_PBI 00233929), 1♂ (AMNH_PBI 00233930) (ZISP) • ***Orenburg Prov.***: Nr Orenburg, 51.76666°N, 55.1°E, 18 Jun 1924, A.I. Ivanov, 1♂ (AMNH_PBI 00233821) (ZISP) • ***Perm Terr.***: Perm, 58.01666°N, 56.3°E, 1925, Lubischev, 1♀ (AMNH_PBI 00233977) (ZISP) • ***Primorsky Terr.***: Tal’bomogi, Tumen’-Ula, Russia-Korean boundary, 42.4144°N, 130.6486°E, 07 Jul 1913, Cherskiy, 1♂ (AMNH_PBI 00233586), 1♀ (AMNH_PBI 00233914) (ZISP) • Vinogradovka, 46.2°N, 134.4°E, 05 Jul 1929, A. N. Kiritshenko, 1♀ (AMNH_PBI 00233926) (ZISP) • 07 Jul 1929, A. N. Kiritshenko, 1♂ (AMNH_PBI 00233813) (ZISP) • 09 Jul 1929, A. N. Kiritshenko, 1♀ (AMNH_PBI 00233927) (ZISP) • Yakovlevka, 44.4°N, 133.45°E, 28 Jun 1926, Dyakonov & Filip’ev, 1♀ (AMNH_PBI 00233939) (ZISP) • ***Rostov Prov.***: Rostov-na-Donu, 47.21666°N, 39.7°E, 29 Jun 1928, Unknown collector, 1♀ (AMNH_PBI 00233960) (ZISP) • ***Samara Prov.***: Krasnaya Glinka, 25 km of Samara, 53.33333°N, 50.18333°E, 08 Jul 1928, Lubischev, 3♀ (AMNH_PBI 00233973-AMNH_PBI 00233975), 2♂ (AMNH_PBI 00233588, AMNH_PBI 00233589) (ZISP) • ***Tambov Prov.***: Michurinsk [Kozlov], 52.88333°N, 40.46666°E, W. H. Lange, 6♂ (AMNH_PBI 00233831, AMNH_PBI 00233584, AMNH_PBI 00233585), 2♀ (AMNH_PBI 00233831) (ZISP) • ***Volgograd Prov.***: Krasnoarmeysk [former Sarepta], 48.5°N, 44.48333°E, V. Jakovlev coll., 2♀ (AMNH_PBI 00233515, AMNH_PBI 00233516) (ZISP) • ***Voronezh Prov.***: Nr Ramon’, 51.91666°N, 39.31666°E, 15 Jun 1984, Golub, 1♀ (AMNH_PBI 00233938) (ZISP) • ***Yakutia Rep.***: Balagannakh, 30 km ESE of Ust’-Nera, 64.498°N, 143.857°E, 04 Jul 1974, N.N. Vinokurov, 3♀ (AMNH_PBI 00233507-AMNH_PBI 00233509) (ZISP) • Batagay on Yana river, NE Yakutia (80 km E Verkhoyansk), 67.65°N, 134.63333°E, 12 Jul 1957, Semenov, 1♂ (AMNH_PBI 00233817), 2♀ (AMNH_PBI 00233935, AMNH_PBI 00233936) (ZISP) • Khokhur-terde on Amga river, 60.7°N, 131.6°E, 05 Aug 1925, Bianchi, 1♀ (AMNH_PBI 00233911) (ZISP) • Left bank of Yana River nr Verkhoyansk, 67.55°N, 133.36666°E, 21 Jul 1974, N.N. Vinokurov, 3♂ (AMNH_PBI 00233572-AMNH_PBI 00233574), 7♀ (AMNH_PBI 00233500-AMNH_PBI 00233506) (ZISP) • ***Yamalo-Nenets Distr.***: Tal’bey on Adz’va River, 68.086°N, 72.048°E, Zhuravskiy, 1♀ (AMNH_PBI 00233915) (ZISP) • ***Yaroslavl Prov.***: Nizhniy Isl., Yaroslavl’ distr., 57.482°N, 40.1°E, 19 Jun 1896, Unknown collector, 7♂ (AMNH_PBI 00233830, AMNH_PBI 00233580-AMNH_PBI 00233582), 6♀ (AMNH_PBI 00233830, AMNH_PBI 00233957, AMNH_PBI 00233958, AMNH_PBI 00233582) (ZISP) • Zhukov Isl., Jaroslavl’ distr., 57.482°N, 40.1°E, 05 Jul 1896, Unknown collector, 1♀ (AMNH_PBI 00233956), 1♂ (AMNH_PBI 00233583) (ZISP). **Ukraine** • Pyatikhatka, Oktyabr’skiy Dist., 45.3°N, 34.26666°E, 28 Jun 1952, Loginova, 9♂ (AMNH_PBI 00233824, AMNH_PBI 00233825, AMNH_PBI 00233806-AMNH_PBI 00233812), 11♀ (AMNH_PBI 00233943-AMNH_PBI 00233950, AMNH_PBI 00233981-AMNH_PBI 00233983) (ZISP) • Near Salgir river, 44.95°N, 34.08333°E, 22 Jun 1924, A. N. Kiritshenko, 1♀ (AMNH_PBI 00233986) (ZISP) • 24 Jun 1924, A. N. Kiritshenko, 3♂ (AMNH_PBI 00233827-AMNH_PBI 00233829), 1♀ (AMNH_PBI 00233951) (ZISP) • 14 Jul 1924, A. N. Kiritshenko, 1♂ (AMNH_PBI 00233826), 2♀ (AMNH_PBI 00233984, AMNH_PBI 00233985) (ZISP) • Cherkasy, 49.436°N, 32.084°E, 18 Jun 1931, Lubischev, 1♂ (AMNH_PBI 00233843) (ZISP) • Izmail, Bessarabiya, 45.35°N, 28.83333°E, 09 Jun 1911, Chernavin, 1♀ (AMNH_PBI 00233925) (ZISP) • Kamyshany [Arnautka] nr Kherson, 46.61666°N, 32.48333°E, 18 May 1939, Nikolaev, 1♂ (AMNH_PBI 00233592) (ZISP) • Korobov on Donets river, 7 km of Zmiev, 49.5889°N, 36.3428°E, 06 Jul 1955–07 Jul 1955, L.V. Arnoldi, 1♀ (AMNH_PBI 00233959) (ZISP) • Korsunskiy monastyr’, cursus inf. fl. Dnepr., 46.7167°N, 33.2167°E, 02 Aug 1928, S. I. Medvedev, 1♀ (AMNH_PBI 00233978) (ZISP) • Kozin [Koncha-Zaspa] nr Kiev, 50.21666°N, 30.63333°E, 12 Jul 1932, S. I. Medvedev, 2♀ (AMNH_PBI 00233988, AMNH_PBI 00233989), 1♂ (AMNH_PBI 00233842) (ZISP) • Odessa, Khadzhib liman, 46.46666°N, 30.71666°E, 09 Jun 1920, A. N. Kiritshenko, 4♀ (AMNH_PBI 00233952-AMNH_PBI 00233954, AMNH_PBI 00233815) (ZISP) • Provalye, 48.16666°N, 39.83333°E, 01 Jul 1947, S. I. Medvedev, 1♂ (AMNH_PBI 00233838) (ZISP) • Stanitsa Luganskaya nr Lugansk, 48.65°N, 39.48333°E, 26 Jul 1927, F.K. Lukjanovitsh, 1♀ (AMNH_PBI 00233992) (ZISP) • Verkhovka [former Mahilyow uezd], 48.9°N, 27.65°E, 10 Jun 1901, Chekini, 1♀ (AMNH_PBI 00233967) (ZISP) • Vilkovo, Izmail Distr., Bessarabiya, 45.406°N, 29.589°E, 30 May 1911, Chernavin, 1♀ (AMNH_PBI 00233987) (ZISP).

##### Diagnosis.

Recognized by the following characters: body oval, total length 3.4–4.0; antennal segment I brown, segment II thin, brown or at least with darkened base and apex (Fig. 4G, K); Color-pattern variable, ranging from uniformly dark brown to pale yellow, with more or less darkened head, pronotum, and endocorium (Fig. 2E–I); dorsum devoid of scale-like setae, clothed exclusively with short, strongly adpressed slivery simple setae (Figs 4L, 6E); apical blades of vesica short and robust, straight, apically diverging (Fig. 7G, H).

*Salicarusroseri* easily differs from congeners by the absence of scale-like setae on dorsum. It further differs by having short, robust, straight, and slightly diverging apical blades of the vesica, being most similar to *S.urnammu* in this respect, although the blades in the latter species are shorter.

##### Redescription.

**Male. *Coloration*.** Highly variable, dorsum ranging from uniformly dark brown to pale yellow, with somewhat darkened head (Fig. 2E, F); pale specimens typically with widely darkened endocorium and partly or entirely dark brown pronotum and scutellum, rarely without any dark markings on dorsum. ***Head***: Entirely dark brown to brown, sometimes with yellow or orange vertex and edging along inner margins of eyes; antennal segment I dark brown to yellow, segment II dark brown to yellow with darkened apex, segments III and IV usually dirty yellow, sometimes brown; labium usually dark brown, pale brown to dirty yellow in the palest specimens. ***Thorax***: Pronotum from uniformly dark or chestnut brown to whitish yellow, frequently with reddish tinge, in pale specimens usually with dark markings on calli and darkened posterior margin, rarely uniformly whitish; scutellum usually dark brown, rarely dirty yellow or orange; hemelytron ranging from uniformly dark brown to whitish yellow, pale specimens typically with entirely yellow or whitish clavus, partly or entirely yellow exocorium, largely darkened endocorium, and yellow or orange cuneus, membrane uniformly dark to pale brown, semitransparent; thoracic pleura usually dark brown, rarely dorsally or entirely yellow; coxae dark brown to brown, femora in dark specimens brown with yellowish apical halves or at least extreme apices, in pale specimens entirely yellow, frequently with reddish tinge, tibiae yellow, tarsi yellow ort apically darkened. ***Abdomen***: Dark brown to yellow.

***Surface and vestiture*.** Dorsum shiny, head and pronotum smooth, scutellum and hemelytron weakly rugose (Fig. 4J–L); clothed with short, strongly adpressed, simple silvery setae, sparse on vertex and pronotum, dense on scutellum and hemelytron; antenna, legs, and abdomen with similar but somewhat longer simple setae; thoracic pleurites with dense, narrow, apically acuminate silvery scale-like setae above scent gland evaporatory area; pronotum with a pair of black erect bristle-like setae at anterior corners; femora with several similar black setae dorso-apically; tibial spines black.

***Structure*.** Body oval, 2.6–2.9× as long as width of pronotum at base, total length 3.6–4.0; head vertical, rather vide, slightly protruding beyond eyes anteriorly and ventrally; vertex flat, posteriorly attenuate and covering anterior margin of pronotum, 1.8–1.9× as wide as eye; antennal segment II at base distinctly thinner than segment I, slightly dilating apically, comparatively short, 0.5–0.6× as long as basal width of pronotum, 0.8–0.9× as long as width of head; pronotum with broadly rounded anterior and posterior corners, 2.0–2.2× as wide as long, 1.5–1.7× as wide as head.

***Genitalia*.** Right paramere elongate-oval, ~ 2.5× as long as wide, with long, straight and apically blunt apical process (Fig. 9P). Left paramere with long and straight apical process, and elongate, comparatively thin, slightly upturned sensory lobe (Fig. 9Q). Vesica large and strongly sclerotized, with straight, short and robust, diverging apical blades (Fig. 7G, H).

**Female. *Coloration, surface and vestiture*.** As in male (Fig. 2G–I). ***Structure*.** Similar to male, body 2.5–2.8× as long as posterior width of pronotum; total length 3.4–3.9; vertex 1.8–2.0× as wide as eye; antennal segment II 0.4–0.5× as long as posterior width of pronotum, 0.7–0.9× as long as width of head; pronotum 2.0–2.3× as wide as long, 1.5–1.6× as wide as head.

***Genitalia*.** Sclerotized rings of dorsal labiate late large, broadly oval (Fig. 10G). Vestibulum thin, S-shaped (Fig. 10H).

##### Distribution.

Widely distributed in the Palearctic, including almost the entire Europe, extending eastward to the Khabarovsk and Kamchatka territories in Russia, and southward to Spain, Italy, Greece, Turkey, Transcaucasia, Iran, Turkmenistan, Kazakhstan, Mongolia, and Inner Mongolia of China. To the north, it extends to the central Fennoscandia, Karelia, Arkhangelsk and Komi Provinces, the southern Yamalo-Nenets district, southern Krasnoyarsk Territory, southern and central Yakutia, and Magadan Territory (Vinokurov et al. 2010; Aukema 2024).

##### Host.

Confined to *Salix* spp. (Southwood and Leston 1959; Kerzhner 1962; Göllner-Scheiding 1974).

#### ﻿ Salicarus urnammu

Taxon classificationAnimaliaHemipteraMiridae

Linnavuori, 1984

A3845B5F-C237-5421-AF6B-CF0420E2DBF2

Figs 3A–D , 4 M–O , 7I, J , 9S–U

Salicarius [sic!] urnammu Linnavuori, 1984: 51.
Salicarus urnammu : Konstantinov (2023): 874 (figures, discussion).

##### Material examined.

**Armenia** • Aralykh, 40.11722°N, 44.27055°E, 07 Jun 1911, K. Satunin, 3♂ (AMNH_PBI 00233861, AMNH_PBI 00233862, AMNH_PBI 00233858) (ZISP). Azerbaijan • Arpa-chay River, 39.4675°N, 44.93444°E, 03 Jul 1937–05 Jul 1937, Ryabov, 1♂ (AMNH_PBI 00233755) (ZISP). **Iran** • ***Ardabil Prov*.**: 10 km W Khalkhal, 37.6179°N, 48.522°E, 08 Jul 2002–09 Jul 2002, R. & S. Linnavuori, 2♀ (ZISP_ENT 00011858, ZISP_ENT 00011859) (NMWC) • Askestan-Site, 1 37°28'N, 48°39'E, 11 Jul 2022, R. Hosseini 4♂ 2♀(UGNHM) • Givi-Khalkhal-Site, 2 37°41'N, 48°30'E, 9 Jul.2022, R. Hosseini, 7♂ 5♀ (UGNHM) • Majareh-Site 3, 37°33'N, 48°36'E, 23 Jul 2022, R. Hosseini 1♀ (UGNHM) • Poonel Khalkhal-Site 3, 37°34'N, 48°39'E 27 Jun 2022, R. Hosseini, 6♂ 3♀ (UGNHM) • ***Guilan Prov.***: Lur-Site 5, 36°51'N, 49°53'E, 13 Jun 2022, R. Hosseini, 1♀ (UGNHM) • Malumeh-Site 1, 36°51'N, 49°55'E, 11 Jun 2022, R. Hosseini, 14♂ 5♀ (UGNHM) • Malumeh-Site 3, 36°51'N, 49°55'E, 11 Jun 2022, R. Hosseini, 8♂ 10♀ (UGNHM) • ***Tehran Prov.***: Shahrestanak, 60 km NE Karaj, 34.8508°N, 50.4544°E, 2100 m, 10 Jul 2005–12 Jul 2005, R. Linnavuori, 2♂ (ZISP_ENT 00011863), 2♀ (ZISP_ENT 00011863) (NMWC). **Iraq** • Sulaymaniyah nr Halabja, 35.555°N, 45.479°E, 11 Jun 1980, R. Linnavuori, 3♂ (ZISP_ENT 00011857, ZISP_ENT 00011860, ZISP_ENT 00011862), 1♀ (ZISP_ENT 00011861) (NMWC), 1♀ (AMNH_PBI 00233754) (ZISP). **Turkmenistan** • Garrygala [Kara-Kala], 38.41666°N, 56.25°E, 20 May 1952, Kryzhanovskij, 1♂ (AMNH_PBI 00233859) (ZISP).

##### Diagnosis.

Recognized by the oval body, total length male 3.5–3.8; female 3.2–3.5; antenna typically yellow, in dark specimens segments I and II partly brown, segment II thin (Fig. 4M, N); Dorsum yellow, frequently with orange tinge, sometimes with partly brown pronotum, scutellum, and endocorium (Fig. 3A–D); vestiture composed of short, strongly adpressed simple silvery setae, dense on scutellum and hemelytron but scarce on vertex and pronotum; hemelytron additionally with scarce, narrow, apically acuminate scale-like setae (Fig. 4O); apical blades of vesica short and robust, straight, apically diverging (Fig. 7I, J).

*Salicarusurnammu* is most similar to *S.concinnus* and *S.roseri* but can usually be distinguished from these species by the color pattern. It further differs from *S.roseri* by the presence of scale-like setae on the hemelytron, and from *S.concinnus* by the diverging apical blades of the vesica. Refer to the diagnoses of these species for additional discussion.

##### Redescription.

**Male. *Coloration*.** Variable, ranging from yellow, frequently with orange tinge, sometimes with partly brown pronotum, scutellum, and endocorium to almost unofrmly dark brown, with yellow base of hemelytron and cuneus (Fig. 3A, B). ***Head***: Orange-yellow, usually with whitish vertex and somewhat darkened clypeus (Fig. 4M, N); in dark specimens dirty yellow, with dark brown clypeus, brown mandibular and maxillary plate, and largely brown frons, sometimes uniformly dark brown; antenna typically yellow, in dark specimens segment I partly or entirely brown, segment II basally and/or apically, sometimes entirely brown; labium orange-yellow to brown, apex of segment IV dark brown. ***Thorax***: Pronotum from yellow to uniformly dark brown, frequently yellow with reddish tinge and brown diffuse spots on calli and darkened posterior margin; scutellum usually orange-yellow, entirely brown in dark specimens; hemelytron usually whitish yellow, usually with large wedge-shaped brown spot occupying entire exocorium except base, in dark specimens entire clavus and corium except base dark brown, cuneus dirty yellow; membrane pale brown, semitransparent; thoracic pleura orange-yellow to dark brown; legs typically orange-yellow, without any color pattern, in dark specimens femora more or less brown, with yellow apices, tibiae with minute spots at bases of tibial spines. ***Abdomen***: Orange-yellow, sometimes with darkened stripes along apical margins of pregenital segments, or uniformly dark brown.

***Surface and vestiture*.** Dorsum shiny, head and pronotum smooth, scutellum and hemelytron weakly rugose; clothed with short, subequal in length to scale-like setae on hemelytron, strongly adpressed, simple silvery setae, scarce on vertex and pronotum, dense on scutellum and hemelytron; hemelytron additionally with scarce, silvery, narrow, apically acuminate scale-like setae (Fig. 4O); thoracic pleurites with scarce silvery scale-like setae above scent gland evaporative area; pronotum with a pair of brown erect bristle-like setae at anterior corners; femora with several similar brown setae dorso-apically; tibial spines black.

***Structure*.** Body oval, 2.8–2.9× as long as posterior width of pronotum, total length 3.5–3.8; head vertical, rather vide, slightly protruding beyond eyes anteriorly and ventrally; vertex flat, posteriorly attenuate and covering anterior margin of pronotum, 1.8–2.1× as wide as eye; antennal segment II at base distinctly thinner than segment I, slightly dilating apically, comparatively short, 0.5–0.6× as long as posterior width of pronotum, 0.8–0.9× as long as width of head; pronotum with broadly rounded anterior and posterior corners, 1.9–2.0× as wide as long, 1.5× as wide as head.

***Genitalia*.** Right paramere elongate-oval, ~ 2.4× as long as wide, with straight, comparatively short, and blunt apical process (Fig. 9S). Right paramere with thin straight apical process and triangular, apically broadly rounded sensory lobe (Fig. 9T). Vesica large, with straight, short and robust, gradually diverging apical blades (Fig. 7G, H).

**Female. *Coloration, surface and vestiture*.** As in male (Fig. 3C, D). ***Structure*.** Similar to male, body 2.5–2.7× as long as posterior width of pronotum; total length 3.2–3.5; vertex 1.9–2.2× as wide as eye; antennal segment II 0.5–0.6× as long as posterior width of pronotum, 0.7–0.9× as long as width of head; pronotum 2.0–2.1× as wide as long, 1.5–1.6× as wide as head.

***Genitalia*.** Sclerotized rings of dorsal labiate plate large, broadly oval.

##### Distribution.

Originally described from Iraq, this species was subsequently found in Turkey, Transcaucasia, Iran, and Turkmenistan (Linnavuori 2007; Konstantinov and Namyatova 2008).

##### Host.

*Salix* spp. (Linnavuori 1984, 2007)

### ﻿*Salicarusfulvicornis* species group

#### ﻿ Salicarus fulvicornis

Taxon classificationAnimaliaHemipteraMiridae

(Jakovlev, 1889)

AF191806-2F32-5E3C-B8C5-F5E182FDE0EF

Figs 1G–I , 4D–F , 6A–C , 7C–D , 9J–L

Agalliastes fulvicornis Jakovlev, 1889: 348.
Chlamydatus fulvicornis : Oshanin (1910): 932 (new comb., catalogue).
Phoenicocoris flagellatus Wagner (1967): 71 (syn. by Kerzhner 1997: 247).
Salicarus fulvicornis : Vinokurov and Kanyukova (1995): 58 (new comb.); Schwartz and Stonedahl (2004): 42, figs. 2, 26 (disc., SEM, MG, host); Lu et al. (2011): 500, fig. 1 (descr., figs); Konstantinov (2023): 874 (phylogenetic placement, figures, discussion).

##### Material examined.

***Lectotype of Agalliastesfulvicornis* Jakovlev, 1889** • ♀ **Mongolia: *Selenge Aimak***: Between Khara and Boroiin [Boro], 48.83°N, 106.195°E, Yakovlev coll. (AMNH_PBI 00233377) (ZISP).

***Paratypes of Phoenicocorisflagellatus*Wagner 1967: Mongolia** • ***Bayan Olgiy Aimak***: Chovd-gol, ~ 15 km E of Ulgij, 49.06666°N, 90.2°E, 1650 m, 28 Jul 1964–29 Jul 1964, Mongolisch - Deutsche Biolog. Exped., 3♂ (AMNH_PBI 00184011, AMNH_PBI 00340326, AMNH_PBI 00340327) (ZMUH).

***Other specimens examined*: Mongolia** • ***Central Aimak***: Nr Songiin [Songino], SW of Ulaanbaatar, steppe, 47.81666°N, 106.66666°E, 18 Jun 1967, I. M. Kerzhner, *Caraganabungei* (Fabaceae), 7♂ (AMNH_PBI 00233373-AMNH_PBI 00233376), 8♀ (AMNH_PBI 00233374, AMNH_PBI 00233447-AMNH_PBI 00233450) (ZISP) • 18 Jun 1967, Zaytsev, 3♂ (AMNH_PBI 00233520, AMNH_PBI 00233521) (ZISP) • 01 Jul 1967, Zaytsev, 3♂ (AMNH_PBI 00233363-AMNH_PBI 00233365), 1♀ (AMNH_PBI 00233432) (ZISP) • 01 Jul 1967, I. M. Kerzhner, *Caraganabungei* (Fabaceae), 13♂ (AMNH_PBI 00233352-AMNH_PBI 00233362, AMNH_PBI 00266431, AMNH_PBI 00266433), 6♀ (AMNH_PBI 00233427-AMNH_PBI 00233431, AMNH_PBI 00266432) (ZISP) • 01 Jul 1967, Emeljanov, 3♂ (AMNH_PBI 00233366-AMNH_PBI 00233368), 5♀ (AMNH_PBI 00233433-AMNH_PBI 00233437) (ZISP) • Nothern mountainside of Bogdo Ula, nr Ulan Bator, 47.81667°N, 107°E, 29 Jun 1967, I. M. Kerzhner, 4♂ (AMNH_PBI 00233369-AMNH_PBI 00233372), 9♀ (AMNH_PBI 00233438-AMNH_PBI 00233446) (ZISP) • ***South Hangay Aimak***: Tuin-Gol river, middle Khalkhin-Gol [Khalkha] river, 45.796°N, 100.807°E, 01 Aug 1926, A. N. Kiritshenko, 7♂ (AMNH_PBI 00233537-AMNH_PBI 00233543), 11♀ (AMNH_PBI 00233456-AMNH_PBI 00233466) (ZISP) • ***Suhbaatar Aimak***: 40 km SE of Barun-Urt, 46.426°N, 113.644°E, 14 Jul 1971, I. M. Kerzhner, 4♂ (AMNH_PBI 00233534, AMNH_PBI 00233535), 4♀ (AMNH_PBI 00233535, AMNH_PBI 00233453) (ZISP) • Dzotol-Khan-Ula Mt., 45.83333°N, 114.66667°E, 12 Jul 1971, I. M. Kerzhner, 1♂ (AMNH_PBI 00233536), 6♀ (AMNH_PBI 00233454, AMNH_PBI 00233455) (ZISP) • Lun-Ula Mt., 30 km WNW of Ovoot [Dariganga], 45.393°N, 113.516°E, 07 Jul 1971, Emeljanov, 6♂ (AMNH_PBI 00233523-AMNH_PBI 00233526), 4♀ (AMNH_PBI 00233524, AMNH_PBI 00233526, AMNH_PBI 00233452) (ZISP) • Mt. Dzun-Nert, 20 km NE of Dariganga, 45.47°N, 114°E, 09 Jul 1971, Emeljanov, 1♂ (AMNH_PBI 00233522), 1♀ (AMNH_PBI 00233451) (ZISP) • Ongon-Els Sands, 15 km SSE Hongor, 45.664°N, 112.819°E, 05 Jul 1971–06 Jul 1971, I. M. Kerzhner, *Caragana* sp. (Fabaceae), 1♂ (AMNH), *Caragana* sp. (Fabaceae), 19♂ (AMNH_PBI 00233527-AMNH_PBI 00233533), 2♀ (AMNH_PBI 00233528, AMNH_PBI 00233529) (ZISP). **Russian Federation** • ***Altai Rep.***: Kosh-Agach, 49.98333°N, 88.63333°E, 08 Jun 1907, N. W. Rodd, 1♀ (AMNH_PBI 00233471) (ZISP) • 05 Jul 1964, I. M. Kerzhner, 25♂ (AMNH_PBI 00233318-AMNH_PBI 00233342), 34♀ (AMNH_PBI 00233385-AMNH_PBI 00233397, AMNH_PBI 00233399-AMNH_PBI 00233419) *Caraganaspinosa* (Fabaceae), 2 larvae (AMNH_PBI 00233343, AMNH_PBI 00233344), 1♀ (AMNH_PBI 00233398) (ZISP) • 10 Jul 1964, I. M. Kerzhner, 2♂ (AMNH_PBI 00233349, AMNH_PBI 00233350), 3♀ (AMNH_PBI 00233423, AMNH_PBI 00233424, AMNH_PBI 00233426) *Caraganaspinosa* (Fabaceae), 1♂ (AMNH_PBI 00233351), 1♀ (AMNH_PBI 00233425) (ZISP) • 22 Jul 1964, I. M. Kerzhner, *Caraganabungei* (Fabaceae), 6♂ (AMNH_PBI 00233312-AMNH_PBI 00233317), 7♀ (AMNH_PBI 00233378-AMNH_PBI 00233384) (ZISP) • 31 Jul 1964, I. M. Kerzhner, 4♂ (AMNH_PBI 00233345-AMNH_PBI 00233348), 3♀ (AMNH_PBI 00233420-AMNH_PBI 00233422) (ZISP) • ***Buryatia Rep.***: Kyakhta [former Troitskosavsk], 50.3508°N, 106.44939°E, 757 m, 27 Jul 1928, F.K. Lukjanovitsh, 4♀ (AMNH_PBI 00233467-AMNH_PBI 00233470) (ZISP).

##### Diagnosis.

Recognized by the following combination of characters: Body in male elongate, almost parallel sized, 3.1–3.6× as long as posterior width of pronotum, total length 3.7–4.0 (Fig. 1I), female more stumpy, 2.4–2.5× as long as posterior width of pronotum, total length 3.1–3.5 (Fig. 1G, H); dorsum uniformly dark brown to brown; antenna pale brown to brown, segment I frequently dirty yellow, segment II thin, rod-shaped; entire dorsum except head clothed with a mixture of narrow, apically acuminate silvery scales and dense, long, ~ 1.5× as long as scales, adpressed simple setae (Fig. 4D–F); vesica small, with long, thin, gradually curving and slightly diverging distally apical blades (Fig. 7C, D).

*Salicarusfulvicornis* is a distinctive species that can be easily distinguished from its congeners. Females of this species may be confused with dark specimens of *S.concinnus* and *S.roseri*. However, *S.fulvicornis* is easily differentiated by the presence of flattened scales on the pronotum and scutellum, as well as by the contrastingly long simple vestiture. It further differs from both species by having long and thin apical blades of the vesica that slightly diverge from each other.

##### Redescription.

**Male. *Coloration*.** Uniformly dark brown to brown (Fig. 1I). ***Head***: Dark brown; antenna pale brown to brown, segment I frequently dirty yellow; labium dark brown with black segment IV. ***Thorax***: Pronotum, scutellum, thoracic pleurites, and hemelytron uniformly dark brown to brown, membrane pale brown, semitransparent; coxae dark brown, femora brown, sometimes with pale brown apices; tibiae pale brown to dirty yellow, with minute dark brown spots at bases of tibial spines; tarsi dirty yellow, apically darkened. ***Abdomen***: Uniformly dark brown.

***Surface and vestiture*.** Dorsum smooth; pronotum, scutellum, and hemelytron clothed with a mixture of silvery, narrow, apically acuminate scale-like setae and dense, long, ~ 1.5× as long as scales, adpressed, goldish yellow simple setae, these setae on corium sometimes dark brown (Fig. 6C); thoracic pleurites densely clothed exclusively with scale-like setae, while vertex antenna, legs, and abdomen covered with goldish yellow simple setae only; tibial spines black.

***Structure*.** Body elongate, almost parallel-sided, 3.1–3.6× as long as posterior width of pronotum; total length 3.7–4.0; head vertical, slightly protruding beyond eyes anteriorly and ventrally; vertex flat, posteriorly distinctly attenuate and covering anterior margin of pronotum, 1.9–2.1× as wide as eye; frons weakly convex; clypeus flat, barely visible in dorsal view; antennal segment II rod-shaped, slightly thinner than segment I, comparatively long, 0.7–0.8× as long as posterior width of pronotum, 1.0–1.1× as long as width of head; pronotum with broadly rounded anterior and posterior corners, 2.0–2.4× as wide as long, 1.5–1.6× as wide as head.

***Genitalia*.** Right paramere elongate-oval, not expanded proximally beyond basal process, with long, straight, apically blunt apical process (Fig. 9J). Right paramere with straight, comparatively short apical process and thin, gradually narrowing, and apically rounded sensory lobe (Fig. 9K). Vesica small, with long, thin, gradually curving and slightly diverging distally apical blades (Fig. 7C, D).

**Female. *Coloration, surface and vestiture*.** As in male (Fig. 1G, H). **Structure.** Similar to male but body shorter, oval, 2.7–3.0× as long as posterior width of pronotum, total length 3.2–3.5; head with slightly more convex frons and clypeus, and with smaller eyes, vertex 2.1–2.4× as wide as eye; antennal segment II distinctly thinner than segment I, shorter than in male, 0.5–0.7× as long as posterior width of pronotum, 0.8–0.9× as long as width of head; pronotum 2.1–2.3× as wide as long, 1.3–1.5× as wide as head.

***Genitalia*.** Dorsal labiate plate with large and broadly oval sclerotized rings.

##### Distribution.

Known from Mongolia, adjacent steppe regions of Russia (Altai Rep., Buryatia Rep., Zabaykalsky Terr.), and Inner Mongolia in China (Kulik 1974; Lu et al. 2011).

##### Hosts.

Feeds on *Caragana* spp. (Fabaceae), including *Caraganabungei* Ledeb. and *Caraganaspinosa* (L.) Vahl ex Hornem.

#### ﻿ Salicarus halimodendri

Taxon classificationAnimaliaHemipteraMiridae

V. G. Putshkov, 1977

6D2B47C0-DAEE-58FD-93E0-7AD44C4D5975

Figs 2A–D , 4G–I , 7E, F , 9M, O , 10F

Salicarus (Salicarus) halimodendri V. G. Putshkov 1977: 367.
Salicarus halimodendri : Konstantinov (2023): 874 (phylogenetic placement, figures, discussion).
Phoenicocoris qiliananus Zheng, 1996 in Zheng and Li (1996: 101). New synonym.
Salicarus qiliananus : Schwartz and Stonedahl (2004): 42 (new combination, discussion, suspected synonymy).

##### Material examined.

***Holotype*: Kazakhstan** • ***East Kazakhstan Prov.***: ♂ Bazarskiy Picket, Zaysan, 47.45°N, 84.86666°E, 22 Jun 1930, F.K. Lukjanovitsh, (AMNH_PBI 00233844) (ZISP).

***Paratypes*: Kazakhstan** • ***Almaty Prov.***: Iliyskiy on Ili River, 43.52194°N, 76.82972°E, 05 Jun 1969, Seitova, 2♂ (AMNH_PBI 00233855) (ZISP) • ***East Kazakhstan Prov.***: Bazarskiy Picket, Zaysan, 47.45°N, 84.86666°E, 22 Jun 1930, F.K. Lukjanovitsh, 3♂ (AMNH_PBI 00233845-AMNH_PBI 00233847), 3♀ (AMNH_PBI 00233993-AMNH_PBI 00233995) (ZISP) • Buran, Mouth of Kaldzhir, 48.01666°N, 85.2°E, 26 Jun 1930, F.K. Lukjanovitsh, *Halimodendronhalodendron* (Fabaceae), 1♀ (AMNH_PBI 00233997) (ZISP) • Burkhatka Picket, Zaysan, 47.45°N, 84.86666°E, 22 Jun 1930, F.K. Lukjanovitsh, 1♀ (AMNH_PBI 00233996) (ZISP) • ***Karaganda Prov.***: 12 km E Balqash [Balkhash], 46.83333°N, 75.1°E, 18 Jun 1962, I. M. Kerzhner, *Halimodendronhalodendron* (Fabaceae), 1♂ (AMNH_PBI 00233854) (ZISP) • ***Kostanay Prov.***: 200 km SO Qyzylorda, nr Tyshkanbay [Akkum], Syt-Darya, 50.06666°N, 62.13333°E, 30 Jun 1966, I. M. Kerzhner, 1♀ (AMNH_PBI 00234005) (ZISP) • ***Kyzylorda Prov.***: 40 km NW of Turkistan, Karatau Mts. Range, 43.562°N, 67.921°E, I. M. Kerzhner, *Halimodendronhalodendron* (Fabaceae), 1♀ (AMNH_PBI 00234000) (ZISP); 18 May 1966–19 May 1966, I. M. Kerzhner, *Halimodendronhalodendron* (Fabaceae), 5♂ (AMNH_PBI 00233848-AMNH_PBI 00233851), 3♀ (AMNH_PBI 00233998, AMNH_PBI 00233999) (ZISP) • 29 May 1966, I. M. Kerzhner, *Halimodendronhalodendron* (Fabaceae), 2♂ (AMNH_PBI 00233852, AMNH_PBI 00233853), 6♀ (AMNH_PBI 00233853, AMNH_PBI 00234001-AMNH_PBI 00234004) (ZISP). **Mongolia** • ***Hovd Aimak***: 15 km S of Bulgan, 45.952°N, 91.564°E, 29 Jul 1970, Narchuk, *Halimodendronhalodendron* (Fabaceae), 1♀ (AMNH_PBI 00234013) (ZISP) • Elhon, 20 km SE Altai on Bodonchi River, 45.68333°N, 92.48333°E, 27 Jul 1970, I. M. Kerzhner, 14♀ (AMNH_PBI 00233856, AMNH_PBI 00234006-AMNH_PBI 00234008, AMNH_PBI 00234010-AMNH_PBI 00234012), 3♂ (AMNH_PBI 00233856, AMNH_PBI 00233857) *Halimodendronhalodendron* (Fabaceae), 3♀ (AMNH_PBI 00234009) (ZISP).

***Other specimens examined*: Kazakhstan** • ***Zhambul Prov.***: Karatau Mts., 4 km S of Karabastau, 42.88722°N, 70.80667°E, 557 m, 18 May 2015, F. Konstantinov & N. Simov, 1♀ (AMNH_PBI 00343015) (ZISP).

##### Diagnosis.

Recognized by the oval body, total length: male 3.6–3.9, female 3.1–3.5; antennal segment I brown, segment II thin, basally or entirely darkened, remining segments dirty yellow (Fig. 4G, H); coloration of dorsum variable, ranging from almost entirely dark brown to pale yellow; dorsum except head with a mixture of narrow, apically acuminate scale-like setae and dense, comparatively long, ~ 1.5× as long as scales, adpressed, silvery simple setae (Fig. 4I); apical blades of vesica very long and thin, gradually curved, abruptly furcate (Fig. 7E, F).

Dark specimens of *Salicarushalimodendri* are somewhat similar to *S.fulvicornis* in having long simple setae and the arrangement of flattened scale-like setae on the dorsum. However, the latter species can be distinguished by the exceptionally long and thin, subapically bifurcate apical blades (Fig. 7C, D).

##### Redescription.

**Male. *Coloration*.** Variable, ranging from almost entirely dark brown to pale yellow (Fig. 2A–D). ***Head***: In dark specimens almost entirely dark brown, with midline on frons and mandibular plate somewhat paler and with vertex always dirty to whitish yellow along posterior margin; in pale specimens head whitish yellow, with a series of brown, frequently confluent lines radiating from midline on frons, entirely or apically brown clypeus, and brown maxillary plate; antennal segment I dark brown to brown, segment II entirely brown to dirty yellow with brown basal one-fourth, remaining segments dirty yellow; labium dirty yellow, with dark brown segment IV. ***Thorax***: Pronotum, scutellum, and hemelytron from uniformly dark brown to whitish yellow, hemelytron in dark specimens usually paler than pronotum; membrane uniformly pale brown to almost colorless; pronotum and scutellum in pale specimens typically with brown markings on calli and on suture between scutellum and mesonotum, sometimes uniformly whitish yellow; coxae usually yellow, rarely paler brown, femora dirty to whitish yellow, with two or three series of large rounded maculae on ventral surfaces and anterior margins, sometimes confluent in dark specimens, and several dark markings at apices of dorsal surfaces; tibiae yellow with minute dark brown spots at bases of tibial spines; thoracic pleurites brown to pale yellow. ***Abdomen***: Brown to pale yellow.

***Surface and vestiture*.** Dorsum weakly rugose, head smooth, shiny. Pronotum, scutellum, and hemelytron with a mixture of silvery scale-like setae and dense, comparatively long, ~ 1.5× as long as scales, adpressed, silvery simple setae; mesopleuron clothed with scale-like setae alone, while vertex, antenna, legs, metapleuron, and abdomen covered exclusively with adpressed silvery simple setae; tibial spines black to dark brown.

***Structure*.** Body oval, 2.7–2.9× as long as posterior width of pronotum; total length 3.6–3.9; head vertical, slightly protruding beyond eyes anteriorly and ventrally; vertex flat, posteriorly distinctly attenuate and covering anterior margin of pronotum, 2.0–2.2× as wide as eye; frons weakly convex; clypeus flat, barely visible in dorsal view; antennal segment II rod-shaped, slightly thinner than segment I, 0.5–0.6× as long as posterior width of pronotum, 0.8–0.9× as long as width of head; pronotum with broadly rounded anterior and posterior corners, 2.3–2.5× as wide as long, 1.4–1.5× as wide as head.

***Genitalia*.** Right paramere elongate-oval, ~ 1.7× as long as wide, with long, slightly narrowing and apically rounded apical process (Fig. 9M). Left paramere with thin and straight apical process and comparatively short, broadly rounded sensory lobe (Fig. 9N). Vesica comparatively large, with very long and thin, gradually curved, abruptly furcate apical blades (Fig. 7E, F).

**Female. *Coloration, surface and vestiture*.** As in male. ***Structure*.** Similar to male but body smaller and more stumpy, 2.4–2.5× as long as posterior width of pronotum, total length 3.1–3.5; head with slightly more convex frons and clypeus, vertex 2.2–2.4× as wide as eye; antennal segment II distinctly thinner than segment I, 0.5× as long as posterior width of pronotum, 0.7–0.8× as long as width of head; pronotum 2.3–2.4× as wide as long, 1.4–1.5× as wide as head.

***Genitalia*.** Sclerotized rings of dorsal labiate plate large, broadly oval. Posterior wall weakly sclerotized, with indistinctly bordered longitudinal sclerotized bands at sides (Fig. 10F).

##### Distribution.

This species inhabits plains and foothills of Central Asia within the area of its host plant, spanning from Uzbekistan and southwestern and southern Kazakhstan to Mongolia.

##### Hosts.

*Salicarushalimodendri* is known to feed exclusively on *Caraganahalodendron* (Pall.) Dum. Cours. (Fabaceae), commonly known as the common salt tree. This distinctive shrub is primarily found in saline deserts and semideserts and was long classified within the monotypic genus *Halimodendron*.

##### Remarks.

*Phoenicocorisqiliananus* Zheng, 1996 was described in Zheng and Li (1996) from Mati in Gansu province, Northwestern China. Schwartz and Stonedahl (2004) transferred this species to *Salicarus* due to the claw and vesica structure and suspected its possible synonymy with *S.halimodendri*, referring to personal communication from I. M. Kerzhner. However, they refrained from formal synonymization pending examination of additional material. Indeed, the coloration of the dorsum, antenna, and legs of *S.qiliananus*, the vestiture composed of short simple setae and narrow, apically acuminate scale-like setae (which are not exclusively restricted to the hemelytron), the structure of both parameres (Zheng and Li 1996: figs 4, 5), and body proportions suggest that this taxon is conspecific with *S.halimodendri*. The only notable distinction is the presence of a single apical blade of the vesica postulated in the original description. However, all *Salicarus* and *Phoenicocoris* species without exception have a twin-coned vesica, while in *S.halimodendri*, the shorter blade is exceptionally short and thin (Fig. 7E, F), and could have been easily overlooked in the aspects chosen by the authors of the original description for making drawings (Zheng and Li 1996: figs 8, 9). Based on the foregoing discussion, we synonymize *Salicarusqiliananus* (Zheng, 1996) with *Salicarushalimodendri* V. G. Putshkov, 1977.

## Supplementary Material

XML Treatment for Salicarus

XML Treatment for Salicarus cavinotum

XML Treatment for Salicarus genistae

XML Treatment for Salicarus nitidus

XML Treatment for Salicarus perpusillus

XML Treatment for Salicarus concinnus

XML Treatment for Salicarus roseri

XML Treatment for Salicarus urnammu

XML Treatment for Salicarus fulvicornis

XML Treatment for Salicarus halimodendri

## ﻿Additional information

### Author ORCIDs

Fedor V. Konstantinov https://orcid.org/0000-0002-7013-5686

Reza Hosseini https://orcid.org/0000-0002-6556-8401

### Data availability

All of the data that support the findings of this study are available in the main text or Supplementary Information.

## Supplementary materials

Supplementary material 1 USI numbers of figured specimens Data type pdf Explanation note Label data. https://binary.pensoft.net/file/1126632This dataset is made available under the Open Database License (http://opendatacommons.org/licenses/odbl/1.0/). The Open Database License (ODbL) is a license agreement intended to allow users to freely share, modify, and use this Dataset while maintaining this same freedom for others, provided that the original source and author(s) are credited.Fedor V. Konstantinov
