# Supplementary material for: ﻿Review of the genus Salicarus (Hemiptera, Heteroptera, Miridae)
Source: Zookeys. 2024 Sep 2;1211:57–100. doi: 10.3897/zookeys.1211.129660 (PMC11384904; doi:10.3897/zookeys.1211.129660)
Supplement: Supplementary material 1 — USI numbers of figured specimens [file zookeys-1211-057_article-129660__-s001.pdf]

Supplementary material 1. USI numbers of figured specimens

Figure	Species	Sex	USI number
1A	*Salicarus concinnus*	male	AMNH_PBI 00233866
1B	*Salicarus concinnus*	male	AMNH_PBI 00233865
1C	*Salicarus concinnus*	male	AMNH_PBI 00233869
1D	*Salicarus concinnus*	female	AMNH_PBI 00233762
1E	*Salicarus concinnus*	female	AMNH_PBI 00233768
1F	*Salicarus concinnus*	female	AMNH_PBI 00233870
1G	*Salicarus fulvicornis*	female	AMNH_PBI 00233427
1H	*Salicarus fulvicornis*	female	AMNH_PBI 00233447
1I	*Salicarus fulvicornis*	male	AMNH_PBI 00233530
2A	*Salicarus halimodendri*	male	AMNH_PBI 00233845
2B	*Salicarus halimodendri*	female	AMNH_PBI 00234006
2C	*Salicarus halimodendri*	female	AMNH_PBI 00234000
2D	*Salicarus halimodendri*	female	AMNH_PBI 00233995
2E	*Salicarus roseri*	male	AMNH_PBI 00233593
2F	*Salicarus roseri*	male	AMNH_PBI 00233834
2G	*Salicarus roseri*	female	AMNH_PBI 00233933
2H	*Salicarus roseri*	female	AMNH_PBI 00233941
2I	*Salicarus roseri*	female	AMNH_PBI 00233953
3A	*Salicarus urnammu*	male	AMNH_PBI 00233861
3B	*Salicarus urnammu*	male	Iran: Malumeh-Site 3, 36°51'24.9"N 49°55'45.2"E, 11 Jun 2022, R. Hosseini
3C	*Salicarus urnammu*	female	AMNH_PBI 00233755
3D	*Salicarus urnammu*	female	AMNH_PBI 00233859
3E	*Salicarus cavinotum*	male	AMNH_PBI 00184019
3F	*Salicarus cavinotum*	female	AMNH_PBI 00336963
3G	*Salicarus genistae*	male	AMNH_PBI 00240949
3H	*Salicarus genistae*	female	AMNH_PBI 00240955
3I	*Salicarus nitidus*	male	AMNH_PBI 00336961
3G	*Salicarus nitidus*	female	AMNH_PBI 00336968
3K	*Salicarus perpusillus*	male	AMNH_PBI 00184020
3L	*Salicarus perpusillus*	female	AMNH_PBI 00336978
4A	*Salicarus concinnus*	male	AMNH_PBI 00233869
4B	*Salicarus concinnus*	female	AMNH_PBI 00233768
4C	*Salicarus concinnus*	female	AMNH_PBI 00233768
4D	*Salicarus fulvicornis*	male	AMNH_PBI 00233367

4E	*Salicarus fulvicornis*	female	AMNH_PBI 00233341
4F	*Salicarus fulvicornis*	male	AMNH_PBI 00233367
4G	*Salicarus halimodendri*	male	AMNH_PBI 00233845
4H	*Salicarus halimodendri*	female	AMNH_PBI 00234012
4I	*Salicarus halimodendri*	male	AMNH_PBI 00233845
4J	*Salicarus roseri*	male	AMNH_PBI 00233574
4K	*Salicarus roseri*	female	AMNH_PBI 00233509
4L	*Salicarus roseri*	female	AMNH_PBI 00233508
4M	*Salicarus urnammu*	male	AMNH_PBI 00233858
4N	*Salicarus urnammu*	female	AMNH_PBI 00233755
4O	*Salicarus urnammu*	female	AMNH_PBI 00233755
5A	*Salicarus cavinotum*	male	AMNH_PBI 00184019
5B	*Salicarus cavinotum*	female	AMNH_PBI 00336963
5C	*Salicarus genistae*	female	AMNH_PBI 00240955
5D	*Salicarus perpusillus*	male	AMNH_PBI 00184020
5E	*Salicarus perpusillus*	female	AMNH_PBI 00336978
5F	*Salicarus perpusillus*	female	AMNH_PBI 00336978
5G	*Salicarus genistae*	male	AMNH_PBI 00240949
5H	*Salicarus nitidus*	male	AMNH_PBI 00336961
5I	*Salicarus nitidus*	female	AMNH_PBI 00336968
8A	*Salicarus cavinotum*	male	AMNH_PBI 00184019
8B-C, H	*Salicarus genistae*	male	AMNH_PBI 00240947
8D-E	*Salicarus nitidus*	male	AMNH_PBI 00336963
8F-G	*Salicarus perpusillus*	male	AMNH_PBI 00336979
8I	*Salicarus nitidus*	male	AMNH_PBI 00336961
9A-B	*Salicarus concinnus*	male	AMNH_PBI 00233864
9C	*Salicarus concinnus*	male	AMNH_PBI 00233869
9D-F	*Salicarus genistae*	male	AMNH_PBI 00240947
9G-I	*Salicarus nitidus*	male	AMNH_PBI 00336961
9J-L	*Salicarus fulvicornis*	male	AMNH_PBI 00233365
9M-O	*Salicarus halimodendri*	male	AMNH_PBI 00233847
9P-R	*Salicarus roseri*	male	AMNH_PBI 00233572
9S-U	*Salicarus urnammu*	male	AMNH_PBI 00233862
10A-B	*Salicarus concinnus*	female	AMNH_PBI 00233757
10C-E	*Salicarus genistae*	female	AMNH_PBI 00336967
10F	*Salicarus halimodendri*	female	AMNH_PBI 00234006
10G-H	*Salicarus roseri*	female	AMNH_PBI 00235500
